# *Shoseiryuto* May Prevent Bronchial Epithelial Tight Junction Disruption by Inhibiting the Inflammatory NF-κB Signaling Pathway

**DOI:** 10.3390/biology15080603

**Published:** 2026-04-11

**Authors:** Jingya Lu, Ailing Hu, Yunhai Lin, Yi Luo, Wenshu Yuan, Takuji Yamaguchi, Zenji Kawakami, Yasushi Ikarashi, Masaaki Abe, Hajime Orita, Hiroyuki Kobayashi

**Affiliations:** 1 Department of Personalized Kampo Medicine, Juntendo University Graduate School of Medicine, Tokyo 113-8421, Japanw.yuan.tg@juntendo.ac.jp (W.Y.); tkyamagu@juntendo.ac.jp (T.Y.); koba@juntendo.ac.jp (H.K.); 2 International Collaborative Research Administration, Juntendo University School of Medicine, Tokyo 113-8431, Japan

**Keywords:** *Shoseiryuto*, glycyrrhizin, isoliquiritigenin, inflammation, bronchial epithelial tight junction, NF-κB signaling pathway

## Abstract

*Shoseiryuto* (SST) is a Kampo medicine widely used to treat respiratory diseases. We previously showed that SST attenuates lipopolysaccharide (LPS)-induced tight junction (TJ) barrier disruption in human bronchial epithelial (16HBE) cells. However, the underlying mechanisms remain unclear. This study aimed to clarify the mechanisms underlying the protective effects of SST. SST attenuated inflammatory responses (increased IL-6 protein and mRNA levels) and TJ disruption (decreased transepithelial electrical resistance, increased sodium fluorescein permeability, and decreased occludin mRNA and protein expression) induced by LPS, hydrogen peroxide (H_2_O_2_), tumor necrosis factor-α (TNF-α), and polyinosinic–polycytidylic acid (Poly I:C). Further analyses using the Poly I:C model confirmed that the effects of SST were comparable to those of the nuclear factor κB (NF-κB) inhibitors SC-514 and BAY11-7085. SST reduced the activation of NF-κB signaling (increased phosphorylation of NF-κB and IκB), similar to BAY11-7085. SST components, isoliquiritigenin (ILQG) and glycyrrhizin (GL), also attenuated inflammation, barrier dysfunction, and NF-κB signaling activity. These findings suggest that (1) activation of the NF-κB signaling pathway might be associated with both inflammatory responses and TJ barrier disruption; (2) SST could reduce these effects, potentially through modulation of NF-κB signaling; and (3) ILQG and GL may contribute, in part, to these activities. Overall, this study provides the first evidence suggesting that SST may exert anti-inflammatory and epithelial barrier-protective effects, possibly via the suppression of the NF-κB signaling pathway.

## Simple Summary

This study investigated the mechanisms responsible for the protective effects of *Shoseiryuto* (SST) on bronchial epithelial tight junction (TJ) barrier dysfunction using cultured human bronchial epithelial cells. SST effectively suppressed inflammation and TJ barrier dysfunction induced by inflammatory agents such as lipopolysaccharide (LPS), hydrogen peroxide (H_2_O_2_), tumor necrosis factor-α (TNF-α), and polyinosinic–polycytidylic acid (Poly I:C). Further analyses were conducted using the Poly I:C model as a representative inflammation model. The protective effects of SST against inflammation and TJ barrier dysfunction were comparable to those of nuclear factor κB (NF-κB) inhibitors and were attributed to the inhibition of NF-κB signaling activation. The SST components isoliquiritigenin (ILQG) and glycyrrhizin (GL) suppressed inflammation, TJ barrier dysfunction, and NF-κB signaling activation, consistent with the effects of SST. These findings suggest that (1) activation of the NF-κB signaling pathway might contribute to both inflammatory responses and TJ barrier disruption, (2) SST could reduce these effects, potentially through the modification of NF-κB signaling, and (3) ILQG and GL may contribute, in part, to these activities. Overall, this study suggests that SST may be a Kampo medicine that possesses both anti-inflammatory and TJ barrier-protecting properties for the treatment of respiratory diseases.

## 1. Introduction

*Shoseiryuto* (SST) is a Kampo formulation approved by the Ministry of Health, Labor and Welfare in Japan for the treatment of allergic rhinitis, bronchial asthma, and common cold symptoms [1]. It is known as Xiao-Qing-Long-Tang (XQLT) in China [2] and *So-Cheong-Ryong-Tang* in Korea [3]. Clinical trials have confirmed the efficacy and safety of SST in patients with allergic rhinitis, bronchial asthma, and atopic dermatitis [3,4]. A recent meta-analysis of 24 randomized controlled trials reported that SST alleviates allergic rhinitis and is both safe and well-tolerated in affected patients [2].

In vivo and in vitro basic studies have shown that SST ameliorates passive cutaneous anaphylaxis, allergic rhinitis, and allergic asthma. The underlying mechanism likely involves the inhibition of histamine release and mast cell degranulation, the suppression of eosinophil proliferation, the inhibition of basophil maturation and differentiation, and the suppression of tumor necrosis factor-α (TNF-α) synthesis in peripheral blood mononuclear cells [5].

In a recent study using cultured human bronchial epithelial (16HBE) cells, we showed that SST exerts protective effects against bronchial epithelial tight junction (TJ) barrier disruption induced by bacterial endotoxin lipopolysaccharide (LPS) [5]. Tight junctions are essential for regulating paracellular permeability and maintaining epithelial barrier integrity against external challenges [6,7]. Therefore, disruption of the TJ barrier is considered a contributing factor to the onset or exacerbation of respiratory diseases, such as asthma and allergic rhinitis [8,9]. These findings suggest that the protective effect of SST against LPS-induced TJ disruption represents a potential mechanism underlying its therapeutic efficacy in respiratory diseases [5]. Given that LPS is a proinflammatory agent [10], we hypothesize that the protective effect of SST on the TJ barrier may result from its anti-inflammatory activity. However, the precise molecular mechanism underlying this effect has not yet been fully clarified.

In addition to LPS, hydrogen peroxide (H_2_O_2_)—an oxidative stress inducer [11]—TNF-α, which is produced by activated macrophages and lymphocytes [12], and polyinosinic–polycytidylic acid (Poly I:C), a double-stranded RNA analog that mimics viral RNA infection [13], are all proinflammatory agents. Although these stimuli activate distinct upstream pathways, many of them can lead to inflammatory cytokine production, such as interleukin (IL)-1β, IL-6, and IL-18, through signaling cascades that involve the activation of nuclear factor κB (NF-κB) [14,15]. Under normal conditions, NF-κB remains inactive in the cytoplasm due to its association with the inhibitory protein inhibitor of κB (IκB). Upon exposure to inflammatory stimuli, the IκB kinase (IKK) complex is activated, resulting in the phosphorylation and degradation of IκB. This process releases NF-κB, allowing for its phosphorylation and activation. Activated NF-κB forms a p50/p65 heterodimer that translocates to the nucleus, binds to the promoter regions of target genes, and regulates their transcription [16,17,18,19]. These mechanisms indicate that NF-κB signaling activation is associated with both the production of inflammatory cytokines and the downregulation of TJ protein expression [20,21]. However, the relationship between NF-κB–mediated inflammation in bronchial epithelial cells and TJ barrier disruption remains unclear.

Therefore, we proposed that NF-κB activation may play a role in mediating both inflammation and TJ disruption in bronchial epithelial cells, and that SST exerts anti-inflammatory and TJ barrier protective effects by inhibiting this signaling pathway.

Based on this hypothesis, the first objective of this study was to elucidate the mechanism by which SST suppresses TJ barrier disruption in cultured 16HBE cells. To achieve this, we first examined the relationship between inflammation and TJ barrier disruption, as well as the effect of SST on both, using inflammation models induced by several proinflammatory agents, including LPS, H_2_O_2_, TNF-α, and Poly I:C. We then compared the effects of SST and NF-κB inhibitors SC-514 and BAY11-7085 on inflammation and TJ barrier disruption, using the Poly I:C model as a representative inflammatory model, to clarify the relationship between the effects of SST and NF-κB signaling activity. To substantiate this relationship, we directly measured the phosphorylation levels of NF-κB and IκB proteins and compared them with those observed after treatment with BAY11-7085.

Through these analyses, we found that the protective effect of SST on TJ barrier disruption was associated with the suppression of NF-κB signaling activity, suggesting that its anti-inflammatory components may contribute to this effect. SST contains numerous anti-inflammatory components, including phenols, flavonoids, and terpenoids [5]. Therefore, as part of our identification of active compounds, the second objective of this study was to explore the effects of isoliquiritigenin (ILQG) and glycyrrhizin (GL) derived from *Glycyrrhiza*, which are readily available and have well-characterized pharmacokinetics [22].

## 2. Materials and Methods

### 2.1. Test Agents

SST dry powder extract (lot no. 2100019010; Tsumura & Co., Tokyo, Japan) was prepared under standardized manufacturing conditions from eight crude drugs in accordance with the Japanese Pharmacopoeia: 6 g of pinellia tuber (*Pinellia ternata* Breitenbach), 3 g of processed ginger (*Zingiber officinale* Roscoe), 3 g of glycyrrhiza (*Glycyrrhiza uralensis* Fischer), 3 g of cinnamon bark (*Cinnamomum cassia* J. Presl), 3 g of schisandra fruit (*Schisandra chinensis* Baillon), 3 g of asiasarum root (*Asiasarum heterotropoides* F. Maekawa var. *mandshuricum* F. Maekawa), 3 g of peony root (*Paeonia lactiflora* Pallas), and 3 g of ephedra herb (*Ephedra sinica* Stapf). The listed crude drug weights correspond to the crude drug equivalents used for preparation of the SST extract. A three-dimensional high-performance liquid chromatography chromatogram of the SST methanol extract has identified 28 components, including terpenoids, flavonoids, phenylpropanoids, tannins, lignans, and catechols [5]. SST extract powder (Tsumura & Co., Tokyo, Japan) was composed of eight crude drugs in accordance with the standardized formulation of the traditional Kampo medicine SST and was not modified. The extraction yield and concentration equivalence of the SST dry extract are defined by the manufacturer’s standardized production process; however, detailed yield percentages were not independently quantified in this study.

ILQG (≥96% purity) and GL (≥90% purity)—two anti-inflammatory components derived from *Glycyrrhiza*—were obtained from Fujifilm Wako Pure Chemical Corporation (Osaka, Japan).

LPS and H_2_O_2_ were sourced from Sigma-Aldrich (St. Louis, MO, USA); TNF-α from Fujifilm Wako (Osaka, Japan); Poly I:C from InvivoGen (San Diego, CA, USA); and the NF-κB inhibitors SC-514 and BAY11-7085 from Abcam (Cambridge, UK) and TargetMol (Boston, MA, USA), respectively.

All test agents were prepared in 0.1% dimethyl sulfoxide to obtain the desired working concentrations.

### 2.2. Cell Culture

A human bronchial epithelial cell line, 16HBE14o–(16HBE cells; 1 × 10^6^ cells), purchased from Merck KGaA (Darmstadt, Germany), was cultured in 75-cm^2^ culture flasks containing 15 mL of minimum essential medium supplemented with 10% fetal bovine serum (FBS; Thermo Fisher Scientific, Waltham, MA, USA) and 1% penicillin–streptomycin solution (Thermo Fisher Scientific). The cells were incubated at 37 °C in a humidified atmosphere with 5% CO_2_. Upon reaching full confluence, defined as greater than 95% cell coverage of the culture surface, as determined under a phase-contrast microscope, the cells were detached using 0.25% trypsin–ethylenediaminetetraacetic acid solution (Thermo Fisher Scientific) and subcultured. Cells at the third passage were used for all experiments.

### 2.3. Experimental Design

In this study, the pharmacological effects of the test substance on inflammation and tight junction disruption were evaluated by administering the test substance simultaneously with inflammatory stimuli. This co-administration was chosen to mimic simultaneous exposure to inflammatory stimuli and therapeutic interventions in clinical or physiological situations. By adopting this paradigm rather than a pre-treatment protocol, the aim was to evaluate the protective effects of the test substance under conditions that more accurately reflect the real-time response to inflammatory challenges.

#### 2.3.1. Effects of Test Agents on Cell Viability

The effects of the test agents, both individually and in combination, on cell viability were evaluated according to a previously described protocol [5]. Briefly, 16HBE cells (5 × 10^3^ cells/well) were plated in 96-well microplates and cultured in 100 μL of medium per well at 37 °C in a humidified atmosphere containing 5% CO_2_. At approximately 80% confluence, defined as ~80% cell coverage of the culture surface, the cells were further incubated for 24 h in medium containing the following compounds, either alone or in combination: SST (0.1–1.0 mg/mL), GL (12.5–100 µM), ILQG (12.5–100 µM), SC-514 (5–25 µM), BAY-11-7085 (1.25–5 µM), LPS (250–1000 µg/mL), H_2_O_2_ (0.1–0.5 mM), TNF-α (75–300 ng/mL), Poly I:C (5–20 µg/mL), LPS (1 mg/mL) + SST (0.1 and 0.3 mg/mL), TNF-α (150 ng/mL) + SST (0.1 and 0.3 mg/mL), H_2_O_2_ (0.5 mM) + SST (0.1 and 0.3 mg/mL), Poly I:C (10 µg/mL) + SST (0.1 and 0.3 mg/mL), Poly I:C (10 µg/mL) + GL (25 µM), Poly I:C (10 µg/mL) + ILQG (25 µM), Poly I:C (10 µg/mL) + SC-514 (5, 10 and 25 µM), and Poly I:C (10 µg/mL) + BAY-11-7085 (1.25, 2.5 and 5 µM).

Cell viability after 24 h of exposure to the test agents was assessed using the method described in Section 2.4.1. These assays were informed by preliminary dose–response experiments conducted to identify concentrations that maintained a detectable biological response without inducing significant cytotoxicity.

#### 2.3.2. Effects of SST on Inflammation and TJ Barrier Disruption

The effects of SST on inflammation and TJ barrier disruption were assessed using four inflammation models induced by LPS, H_2_O_2_, TNF-α, and Poly I:C. These effects were evaluated by measuring inflammatory markers (IL-6 levels in the culture medium and its intracellular mRNA expression) and TJ barrier markers [transepithelial electrical resistance (TEER), sodium fluorescein (Na–F) permeability of cultured cell monolayers, and the mRNA and protein expression of the TJ protein occludin]. The experiments were performed using a liquid–liquid interface culture system in a transwell chamber divided into apical (360 μL) and basolateral (1.3 mL) compartments using an insert basket [5].

LPS model: 16HBE cells (1 × 10^5^ cells/well) were seeded onto the membrane inserts of 24-well transwell plates filled with standard medium in both compartments and incubated at 37 °C under 5% CO_2_ for 3 days. Once the cells formed a confluent monolayer, the medium in both compartments was replaced with either standard medium (control) or medium containing LPS (1 mg/mL), alone or in combination with SST (0.1 or 0.3 mg/mL). The cells were then incubated for an additional 48 h, after which inflammatory and TJ barrier markers were measured, as described in Section 2.4.2 and Section 2.4.3.

H_2_O_2_ model: Following the same culture procedure as the LPS model, 16HBE cells were cultured for 3 days to form a confluent monolayer. The cells were then divided into three groups: control, H_2_O_2_, and H_2_O_2_ + SST. Treatments were conducted as follows: the control group was incubated in standard medium for 30 min and subsequently for 24 h in the same medium; the H_2_O_2_ group was exposed to 0.5 mM H_2_O_2_ for 30 min and then cultured for 24 h in normal medium; and the H_2_O_2_ + SST group was exposed to 0.5 mM H_2_O_2_ for 30 min, followed by 24 h in SST-containing medium (0.1 or 0.3 mg/mL). Inflammatory and TJ barrier markers were then measured using the assay methods described in Section 2.4.2 and Section 2.4.3.

TNF-α and Poly I:C models: Following the same procedure as the LPS model, 16HBE cells were cultured for 3 days to form a confluent monolayer. The cells were then divided into control, TNF-α (or Poly I:C), and TNF-α (or Poly I:C) + SST groups and treated as follows: the control group was cultured in standard medium for 24 h; the TNF-α (or Poly I:C) group was cultured in medium containing 150 ng/mL TNF-α (or 10 µg/mL Poly I:C) for 24 h; and the TNF-α (or Poly I:C) + SST group was cultured in medium containing 150 ng/mL TNF-α (or 10 µg/mL Poly I:C) + SST (0.1 mg/mL and 0.3 mg/mL) for 24 h. Inflammatory and TJ barrier markers were then measured using the assay methods described in Section 2.4.2 and Section 2.4.3.

The concentrations and exposure durations of the inflammatory stimuli were selected based on previous reports [5,23] and preliminary optimization experiments to induce measurable inflammatory responses while minimizing cytotoxic effects.

Specifically, H_2_O_2_ was applied for 30 min, followed by replacement with fresh medium to avoid excessive cytotoxicity while inducing oxidative stress, whereas LPS, TNF-α, and Poly I:C were applied for 48 h and 24 h to allow for sufficient activation of inflammatory signaling pathways.

#### 2.3.3. Effects of NF-κB Signaling Inhibitors on Inflammation and TJ Barrier Disruption

Experiments were performed using the Poly I:C model described in Section 2.3.2, with minor modifications. Briefly, 16HBE cells were cultured for 3 days to form a confluent monolayer, after which they were divided into four groups: control, NF-κB signaling inhibitor (SC-514 or BAY11-7085), Poly I:C, and Poly I:C + NF-κB signaling inhibitor groups. These inhibitors target different stages of NF-κB activation: SC-514 inhibits IKK activity, while BAY-11-7085 prevents IκB phosphorylation. The treatment protocols were as follows: the control group was cultured in standard medium for 2 h, followed by an additional 24 h in the same medium; the NF-κB signaling inhibitor group was cultured in medium containing SC-514 (10 μM) or BAY11-7085 (5 μM) for 2 h, then transferred to standard medium for 24 h; the Poly I:C group was cultured in standard medium for 2 h, followed by 24 h in Poly I:C (10 µg/mL)-supplemented medium; and the Poly I:C + NF-κB signaling inhibitor group was cultured in SC-514 (10 μM) or BAY11-7085 (5 μM)-containing medium for 2 h, followed by 24 h in Poly I:C (10 µg/mL)-supplemented medium. Inflammatory and TJ barrier markers were subsequently quantified using the methods described in Section 2.4.2 and Section 2.4.3.

#### 2.3.4. Effects of SST and BAY11-7085 on the Phosphorylation Levels of NF-κB p65 and IκBα Proteins

The experiments evaluating the effects of SST in the Poly I:C model were conducted under the same experimental conditions and schedule described in Section 2.3.2. The experimental group included control, SST (0.3 mg/mL), Poly I:C (10 µg/mL), and Poly I:C (10 µg/mL) + SST (0.3 mg/mL). Similarly, experiments examining the effect of the NF-κB signaling inhibitor BAY11-7085 in the Poly I:C model were performed under the same experimental conditions and schedule as described in Section 2.3.3. The groups consisted of the control, BAY11-7085 (5 µM), Poly I:C (10 µg/mL), and Poly I:C (10 µg/mL) + BAY11-7085 (5 µM). Phosphorylation levels of NF-κB p65 and IκBα proteins in the control and treated cells were measured using the assay method described in Section 2.4.4.

#### 2.3.5. Effects of GL and ILQG on Poly I:C-Induced Inflammation, TJ Barrier Disruption, and NF-κB Signaling Activity

The experiments evaluating the effects of ILQG and GL in the Poly I:C model were conducted under the same experimental conditions and schedule as those used for SST evaluation in the same model (see Section 2.3.2). The group included control, Poly I:C (10 μg/mL), Poly I:C (10 μg/mL) + ILQG (25 µM) or GL (25 µM). Markers of inflammation, TJ barrier integrity, and NF-κB signaling activity in control and treated cells were measured using the assays described in Section 2.4.2, Section 2.4.3 and Section 2.4.4.

### 2.4. Assay Methods

#### 2.4.1. Measurements of Cell Viability

Cell viability following exposure to the test agents was determined using a CCK-8 assay kit (Dojindo Molecular Technologies, Gaithersburg, MD, USA) according to the manufacturer’s instructions. Absorbance was measured at 450 nm with a microplate reader (Thermo Fisher Scientific), as described in our previous report [5]. The viability of the treated cells was expressed as a percentage of the absorbance measured in untreated control cells.

#### 2.4.2. Measurement of Inflammatory Markers

IL-6 Protein Concentration: The concentration of IL-6 in the culture medium was determined using a human IL-6 ELISA kit (Chondrex Inc., Redmond, WA, USA) according to the manufacturer’s instructions, as described in our previous report [24].

IL-6 mRNA Expression: The expression of IL-6 mRNA in cells was analyzed using real-time quantitative polymerase chain reaction (RT-qPCR), following a previously described procedure [5]. Briefly, total RNA was extracted using the RNeasy Plus Mini Kit (Qiagen LLC, Germantown, MD, USA), and reverse transcription from RNA to cDNA was performed using the ReverTra Ace^®^ qPCR RT Kit (Toyobo Co., Ltd., Tokyo, Japan). RT-qPCR was performed with the Applied Biosystems 7500 Fast Real-Time PCR System (Thermo Fisher Scientific, Waltham, MA, USA) using KOD SYBR^®^ qPCR Mix (Toyobo Co., Ltd., Tokyo, Japan). The primers used were specific to the target gene *IL-6* and the endogenous control gene *glyceraldehyde-3-phosphate dehydrogenase* (*GAPDH*). The sequences of the forward (F) and reverse (R) primers were as follows:

*IL-6*: (F) 5′-TCCACAAGCGCCTTCGGTCC-3′ and (R) 5′-GTGGCTGTCTGTGTGGGGCG-3′; *GAPDH*: (F) 5′-GACCTGACCTGCCGTCTA-3′ and (R) 5′-GTTGCTGTAGCCAAATTCGTT-3′. The relative IL-6 mRNA expression level was normalized to *GAPDH* expression and expressed as fold change relative to the control (set as 1.0). The concentration of the secreted pro-inflammatory cytokine IL-6 in the culture medium was measured as an indicator of the inflammatory response.

#### 2.4.3. Measurement of TJ Barrier Markers

TEER: TEER was measured using an EVOM3 epithelial volt-ohm meter (World Precision Instruments, Sarasota, FL, USA) equipped with STX2-plus “chopstick” electrodes, following the procedure described in our previous report [5]. Changes in TEER following treatment with the test agents were expressed as percentages relative to the control values. TEER between cell monolayers has been reported to increase with TJ formation and decrease when barrier integrity is compromised [5].

Sodium Fluorescein (Na–F) Permeability: The concentration of Na–F used to assess apical-to-basolateral permeability across the monolayer barrier was measured using a fluorometer (Thermo Fisher Scientific) according to the procedures described in our previous report [5]. Na–F permeability was calculated as follows:

Na–F permeability (%) = (Na–F_basolateral_/Na–F_apical_) × 100

where Na–F_apical_ is the amount of Na–F added to the apical medium as an index of permeability, and Na–F_basolateral_ is the amount of Na–F transferred to the basolateral medium 1 h after addition. The intercellular permeability of Na–F is known to decrease with TJ barrier formation and increase when the barrier weakens [5].

Occludin Protein: Intracellular occludin protein was detected by immunofluorescence staining, as previously reported [5]. Briefly, cells treated or untreated with test agents were stained with anti-occludin antibody (1:100; Santa Cruz Biotechnology, Dallas, TX, USA), followed by nuclear staining with 4′,6-diamidino-2-phenylindole. The samples were then examined under a fluorescence microscope (BZ-X700, Keyence, Osaka, Japan) equipped with a 20× objective lens. All images were captured with identical exposure settings. Other than uniform brightness and contrast adjustment, no post-acquisition processing was performed.

Occludin mRNA Expression: Intracellular occludin mRNA expression was quantified using RT-qPCR, following the same procedure used for IL-6 mRNA analysis. The primer sequences for occludin were as follows:

(F) 5′-GACACTGGCCTACAGGAATACA-3′ and (R) 5′-ATTCATCAGCAGCAGCCATGT-3′. Occludin mRNA expression levels were normalized to the expression level of the *GAPDH* gene, used as the endogenous control, and expressed relative to the control (set as 1.0).

#### 2.4.4. Measurement of NF-κBα Signaling Pathway Markers

Protein levels of NF-κB (p65), IκBα, and their phosphorylated forms (P-p65 and P-IκBα), as indicators of NF-κB signaling pathway activation, were measured using Western blot analysis according to previously reported procedures [25]. Briefly, total cellular proteins were extracted from cells using a RIPA lysis buffer. Protein concentrations were determined using a bicinchoninic acid protein assay kit (Thermo Fisher Scientific). Equal amounts of protein (7–10 μg/lane) were separated by 15% sodium dodecyl sulfate–polyacrylamide gel electrophoresis and transferred to polyvinylidene fluoride membranes, which were blocked with 5% bovine serum albumin (Sigma-Aldrich) to prevent nonspecific binding and then incubated overnight at 4 °C with rabbit monoclonal antibodies (Cell Signaling Technology, Danvers, MA, USA) against NF-κB p65 (#4764; 1:1000), phospho-NF-κB p65 (#3033; 1:1000), IκBα (#9242; 1:1000), phospho-IκBα (#2859; 1:1000), and β-actin (#5125; 1:10,000), washed, and then incubated for 1 h at room temperature with horseradish peroxidase-conjugated secondary antibodies (1:100; #7074, Cell Signaling Technology). Protein bands were visualized using enhanced chemiluminescence reagent (GE Healthcare, Chicago, IL, USA), and densitometric analysis was performed using ImageJ software version 1.53k (National Institutes of Health, Bethesda, MD, USA). β-actin was used as a loading control to normalize protein levels. Phosphorylated and total proteins were detected on parallel membranes using the same protein lysates, and β-actin was probed separately for each membrane. Phosphorylation of the p65 and IκBα proteins was quantified as the ratio of P-p65/p65 and P-IκBα/IκBα, respectively. Whole-cell lysates were used for Western blot analysis.

### 2.5. Statistical Analysis

All data are presented as the mean ± standard deviation (SD). The normality of the data distribution was assessed using the Shapiro–Wilk test prior to statistical analysis. Statistical significance was determined using a one-way ANOVA, followed by Dunnett’s post hoc multiple comparisons test. A *p*-value of <0.05 was considered statistically significant. All analyses were conducted using GraphPad Prism version 9 (GraphPad Software, San Diego, CA, USA).

## 3. Results and Discussion

### 3.1. Cytotoxicity

**Figure 1:**
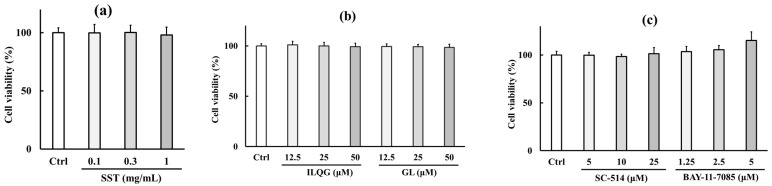

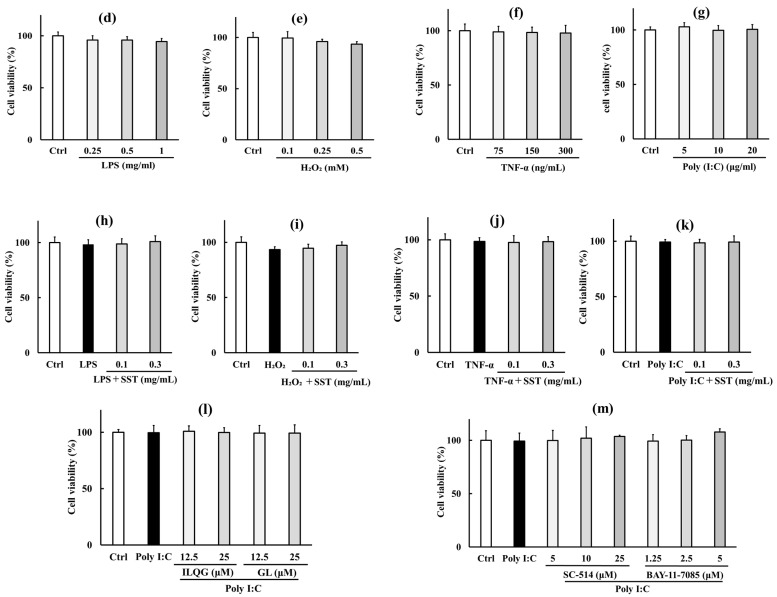
Cell viability of 16HBE cells treated with individual and combined concentrations of test agents. (**a**) SST, (**b**) ILQG, and GL derived from *Glycyrrhiza*, a component crude drug of SST, (**c**) NF-κB signaling inhibitors SC-514 and BAY-11-7085, (**d**–**g**) four proinflammatory agents (**d**) LPS, (**e**) H_2_O_2_ (**f**) TNF-α, and (**g**) Poly I:C, and in combination with inflammatory inducers (**h**) 1 mg/mL LPS + SST, (**i**) 0.5 mM H_2_O_2_ + SST, (**j**) 150 ng/mL TNF-α + SST, (**k**) 10 μg/mL Poly I:C + SST, (**l**) 10 μg/mL Poly I:C + ILQG or GL, and (**m**) 10 μg/mL Poly I:C + SC-514 or BAY-11-7085. Data are presented as mean ± SD (*n* = 3). No significant differences were observed between the control (Ctrl) and treated groups (one-way ANOVA with Dunnett’s test).

To accurately evaluate the specific effects of test agents in cell culture experiments, it is essential to avoid concentrations that induce cytotoxicity. The preliminary experiments confirmed that SST, GL, ILQG, SC-514, BAY-11-7085, LPS, H_2_O_2_, TNF-α, and Poly I:C alone reduced cell viability at concentrations of >1.5 mg/mL, >100 μM, >100 μM, >100 μM, >10 μM, >1.5 mg/mL, >0.5 mM, >1000 ng/mL, and >50 μg/mL, respectively. Figure 1 shows the viability of cells treated with individual test agents and their combinations at the concentrations used in the experimental designs. Cell viability was not significantly different between untreated controls and cells treated with SST, GL, ILQG, SC-514, BAY-11-7085, LPS, H_2_O_2_, TNF-α, or Poly I:C, either alone or in combination with inflammatory inducers (LPS + SST, TNF-α + SST, H_2_O_2_ + SST, Poly I:C + SST, Poly I:C + GL, Poly I:C + ILQG, Poly I:C + SC-514, or Poly I:C + BAY-11-7085). These findings indicate that the cellular responses observed across all experimental conditions reflect the pharmacological effects of the test agents rather than nonspecific cytotoxicity.

### 3.2. Protective Effects of SST on Inflammation and TJ Barrier Disruption

**Figure 2:**
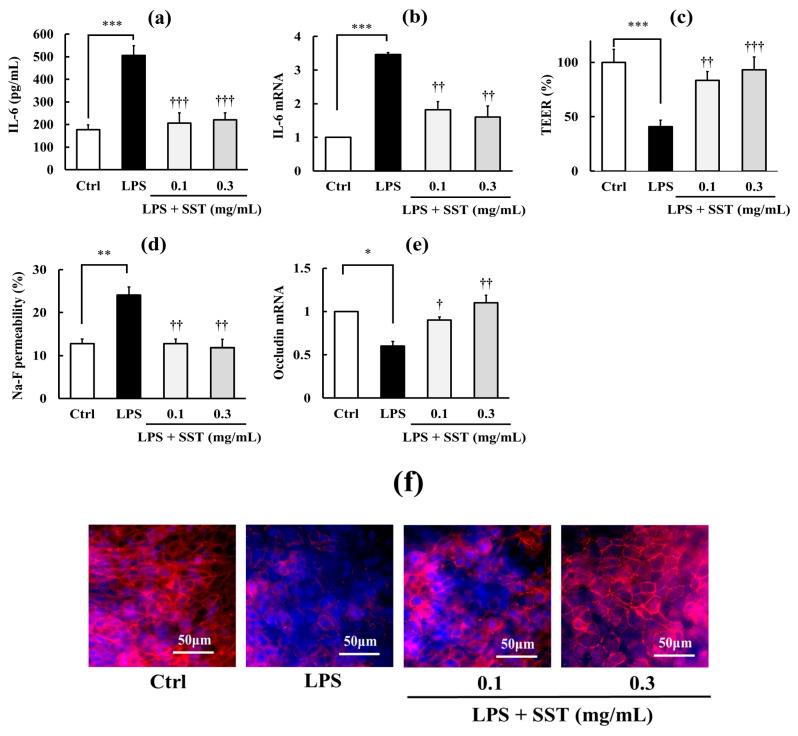
Protective effects of SST on LPS-induced inflammation and TJ barrier disruption. (**a**,**b**) Inflammatory markers: medium IL-6 concentration and IL-6 mRNA expression. (**c**–**f**) TJ barrier markers: TEER, Na–F permeability, occludin mRNA expression, and representative immunofluorescence images of occludin localization. Occludin is shown in red and nuclei are stained with DAPI (blue). LPS concentration: 1 mg/mL. Data are presented as mean ± SD (*n* = 3). * *p* < 0.05, ** *p* < 0.01, *** *p* < 0.001 versus Ctrl; ^†^ *p* < 0.05, ^††^ *p* < 0.01, ^†††^ *p* < 0.001 versus LPS (one-way ANOVA with Dunnett’s test).

Figure 2 shows the protective effects of SST against inflammation and TJ barrier disruption induced by LPS. LPS (1 mg/mL) significantly increased (*p* < 0.001) both the IL-6 concentration in the culture medium and its intracellular mRNA expression (Figure 2a,b), indicating the induction of an inflammatory response under these conditions. Simultaneously, LPS disrupted the TJ barrier, as evidenced by a reduction in TEER values (*p* < 0.001), an increase in Na–F permeability (*p* < 0.01), and decreased occludin mRNA (*p* < 0.05) and protein expression (Figure 2c–f). Cotreatment with SST (0.1 and 0.3 mg/mL) significantly attenuated LPS-induced inflammation and TJ barrier disruption (Figure 2a–f), corroborating our previous findings [5]. Although the relationship between inflammation and TJ barrier dysfunction remains incompletely understood, these findings suggest a close relationship between these two processes. Although not shown in the figure, SST alone (0.1 and 0.3 mg/mL) had no significant effect on any of the basal parameters (TEER, or Na-F permeability, occludin mRNA), which is consistent with our previous report [5].

The LPS concentration (1 mg/mL) was selected based on previous studies using similar epithelial models [5]. This relatively high concentration was necessary because 16HBE cells exhibit lower sensitivity to LPS compared to immune cells [5]; preliminary experiments confirmed that this dose induces a consistent inflammatory response without significant cytotoxicity.

**Figure 3:**
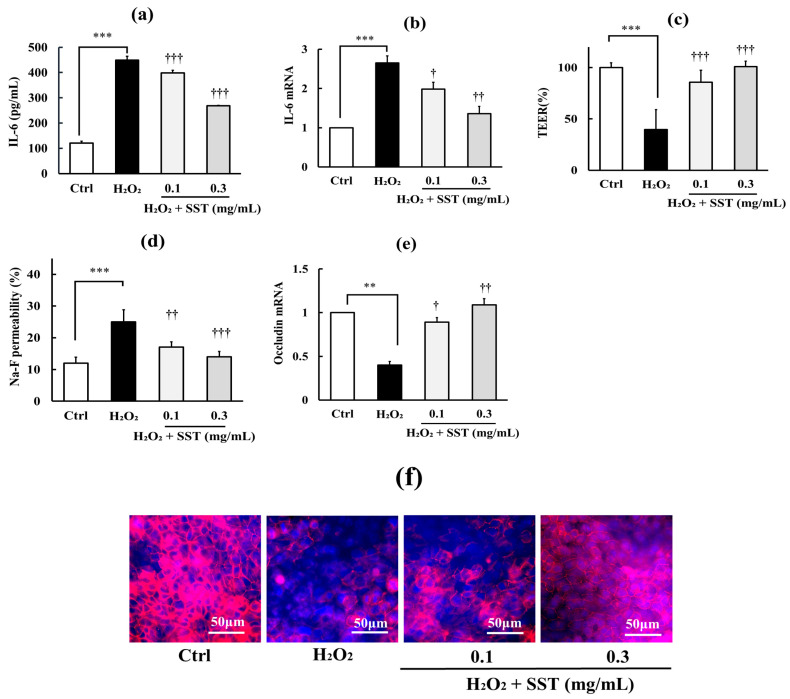
Protective effects of SST on H_2_O_2_-induced inflammation and TJ barrier disruption. (**a**,**b**) Inflammatory markers: medium IL-6 concentration and IL-6 mRNA expression. (**c**–**f**) TJ barrier markers: TEER, Na–F permeability, and occludin mRNA expression and representative immunofluorescence images of occludin localization. Occludin is shown in red and nuclei are stained with DAPI (blue). H_2_O_2_ concentration: 0.5 mM. Data are presented as mean ± SD (*n* = 3). ** *p* < 0.01, *** *p* < 0.001 versus Ctrl; ^†^ *p* < 0.05, ^††^ *p* < 0.01, ^†††^ *p* < 0.001 versus H_2_O_2_ (one-way ANOVA with Dunnett’s test).

**Figure 4:**
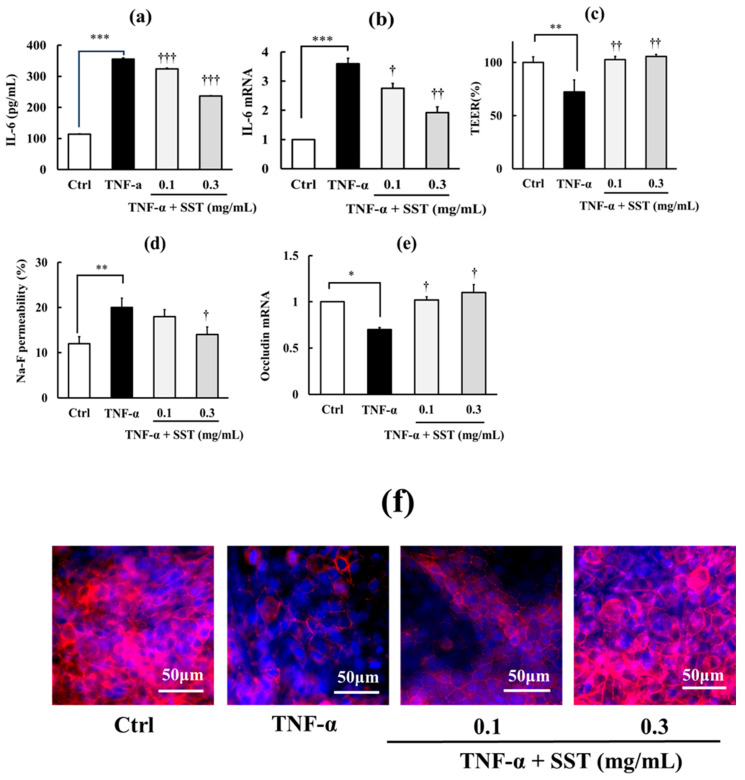
Protective effects of SST on TNF-α-induced inflammation and TJ barrier disruption. (**a**,**b**) Inflammatory markers: medium IL-6 concentration and IL-6 mRNA expression. (**c**–**f**) TJ barrier markers: TEER, Na–F permeability, occludin mRNA expression, and representative immunofluorescence images of occludin localization. Occludin is shown in red and nuclei are stained with DAPI (blue). The TNF-α concentration used in the experiment was 150 ng/mL. Data are presented as mean ± SD (*n* = 3). * *p* < 0.05, ** *p* < 0.01, *** *p* < 0.001 versus Ctrl; ^†^ *p* < 0.05, ^††^ *p* < 0.01, ^†††^ *p* < 0.001 versus TNF-α (one-way ANOVA with Dunnett’s test).

**Figure 5:**
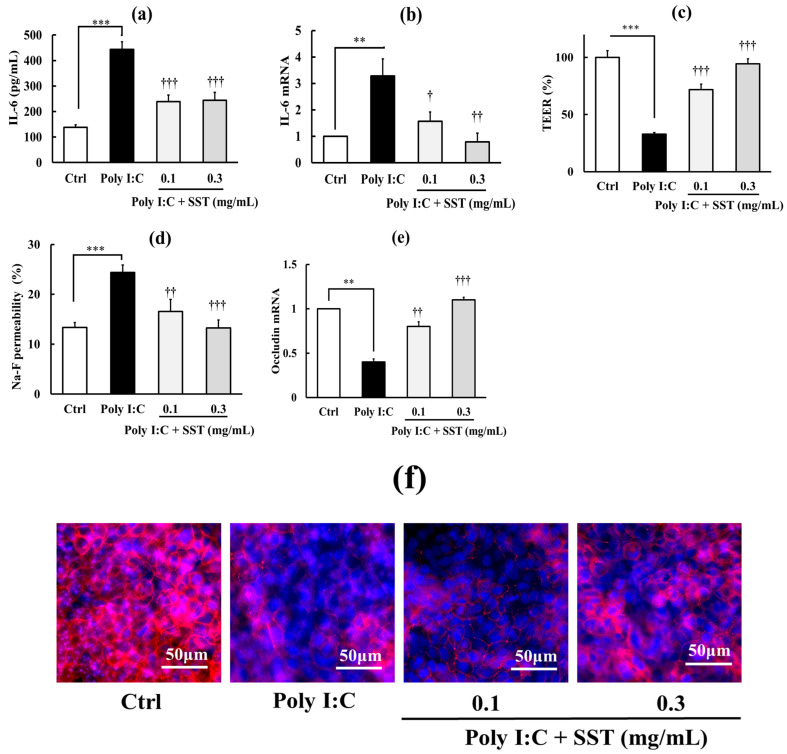
Protective effects of SST on Poly I:C-induced inflammation and TJ barrier disruption. (**a**,**b**) Inflammatory markers: medium IL-6 concentration and IL-6 mRNA expression. (**c**–**f**) TJ barrier markers: TEER, Na–F permeability, occludin mRNA expression, and representative immunofluorescence images of occludin localization. Occludin is shown in red and nuclei are stained with DAPI (blue). Poly I:C concentration: 10 µg/mL. Data are presented as mean ± SD (*n* = 3). ** *p* < 0.01, *** *p* < 0.001 versus Ctrl; ^†^ *p* < 0.05, ^††^ *p* < 0.01, ^†††^ *p* < 0.001 versus Poly I:C (one-way ANOVA with Dunnett’s test).

To further clarify this relationship, we assessed whether proinflammatory agents with distinct mechanisms of action—H_2_O_2_, TNF-α, and Poly I:C—produce similar effects. As shown in Figure 3, Figure 4 and Figure 5 (a–f in each Figure), H_2_O_2_ (0.5 mM), TNF-α (150 ng/mL), and Poly I:C (10 μg/mL) all induced inflammation, indicated by significant increases in IL-6 concentration in the culture medium (*p* < 0.001) and its intracellular mRNA expression (*p* < 0.01–0.001), and TJ barrier disruption, indicated by significantly decreased TEER values (*p* < 0.01–0.001), increased Na–F permeability (*p* < 0.01–0.001), and reduced occludin mRNA (*p* < 0.05–0.01) and protein expression levels. SST (0.1 and 0.3 mg/mL) significantly reduced these effects in a dose-dependent manner (*p* < 0.05–0.001). Although not shown in the figures, SST alone (0.1 and 0.3 mg/mL) had no significant effect on any basal parameters under these experimental conditions.

Mechanistically, LPS induces inflammation by activating TLR4 expressed on the cell surface [26], H_2_O_2_ by inducing oxidative stress within the cell [27], TNF-α by activating TNF receptors on the cell surface [28], and Poly I:C by activating TLR3 in endosomes [29]. Although these diverse stimuli activate distinct upstream signaling cascades, NF-κB acts as a common downstream signaling pathway in many contexts that regulates the expression of pro-inflammatory genes, including cytokines and TJ-related proteins [26,27,28,29].

Given that each proinflammatory agent in this study significantly increased IL-6 mRNA expression (*p* < 0.05–0.001) while decreasing the mRNA expression of the TJ protein occludin (*p* < 0.05–0.001), our findings indicate that NF-κB signaling is associated with both inflammatory response and TJ barrier disruption. Since SST demonstrated consistent efficacy across four models involving distinct receptors (TLR4 for LPS, TLR3 for Poly I:C, and TNFR for TNF-α), we propose that the mechanism likely involves the downstream NF-κB pathway shared by these models, rather than direct interference with receptor–ligand binding.

It should be noted, however, that while NF-κB is a central mediator in these processes, it acts as a common downstream pathway in many, but not all, biological contexts. For instance, certain inflammatory responses and TJ alterations can be driven by other signaling cascades, such as the mitogen-activated protein kinase (MAPK) /activator protein-1 (AP-1) or Janus kinase (JAK)/signal transducer and activator of transcription (STAT) pathways, either independently of or in crosstalk with NF-κB [30,31]. Therefore, the involvement of these alternative pathways remains a limitation of the present study, as discussed below.

### 3.3. Involvement of the NF-κB Signaling Pathway in Inflammation and TJ Barrier Disruption

To determine whether the NF-κB signaling pathway mediates TJ barrier disruption, we employed the Poly I:C-induced inflammation model as a representative system and assessed the effects of two NF-κB signaling pathway inhibitors, SC-514 and BAY11-7085, on Poly I:C-induced inflammation and TJ barrier disruption. SC-514 suppresses NF-κB activation by directly inhibiting IKKβ activity. In contrast, BAY11-7085 suppresses NF-κB activation by inhibiting the phosphorylation of IκBα, thereby preventing IκBα degradation. Consequently, nuclear translocation of NF-κB is blocked due to inhibition of the signaling pathway. Concentrations of 10 μM SC-514 and 5 μM BAY11-7085 have been shown to inhibit NF-κB signaling through these mechanisms [32,33].

**Figure 6:**
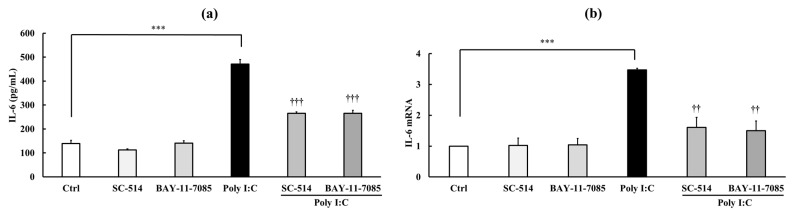

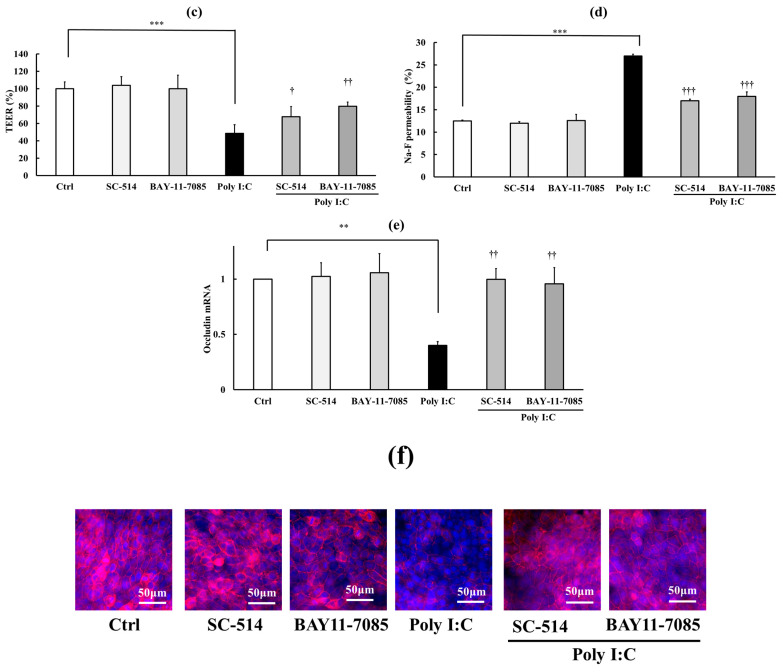
Effects of NF-κB signaling inhibitors SC-514 and BAY11-7085 on Poly I:C-induced inflammation and TJ barrier disruption. (**a**,**b**) Inflammatory markers: IL-6 medium concentration and IL-6 mRNA expression. (**c**–**f**) TJ barrier markers: TEER, Na–F permeability, occludin mRNA expression, and immunofluorescent-stained protein. Occludin is shown in red and nuclei are stained with DAPI (blue). Data are presented as mean ± SD (*n* = 3). ** *p* < 0.01, *** *p* < 0.001 versus Ctrl; ^†^ *p* < 0.05, ^††^ *p* < 0.01, ^†††^ *p* < 0.001 versus Poly I:C (one-way ANOVA with Dunnett’s test).

As shown in Figure 6a–f, SC-514 (10 μM) and BAY11-7085 (5 μM) did not affect baseline inflammation or TJ barrier integrity. However, when combined with Poly I:C (10 μg/mL), both inhibitors significantly suppressed Poly I:C-induced inflammation (increased medium and mRNA expression levels of the inflammatory cytokine IL-6: *p* < 0.001) and TJ barrier disruption (decreased TEER values: *p* < 0.001, increased Na–F permeability: *p* < 0.001, and decreased occludin mRNA *p* < 0.01 and protein expression). These effects were comparable to those observed with SST (Figure 5a–f), supporting the interpretation that the anti-inflammatory and TJ barrier-protective effects of SST may be mediated, at least in part, through the inhibition of NF-κB signaling, similar to the effects of SC-514 and BAY11-7085.

### 3.4. Inhibitory Effect of SST on the NF-κB Signaling Pathway

In normal cells without inflammatory stimuli, NF-κB remains inactive in the cytoplasm due to its association with the inhibitor protein IκB. Upon stimulation with inflammatory agents such as Poly I:C, TNF-α, or LPS, the IKK complex is activated, resulting in the phosphorylation of IκB and NF-κB. Phosphorylated IκB is ubiquitinated and degraded by the proteasome, releasing NF-κB from its binding with IκB, which becomes phosphorylated and activated. Activated NF-κB translocates to the nucleus and functions as a transcription factor that regulates the expression of target genes [16,19,34,35].

**Figure 7:**
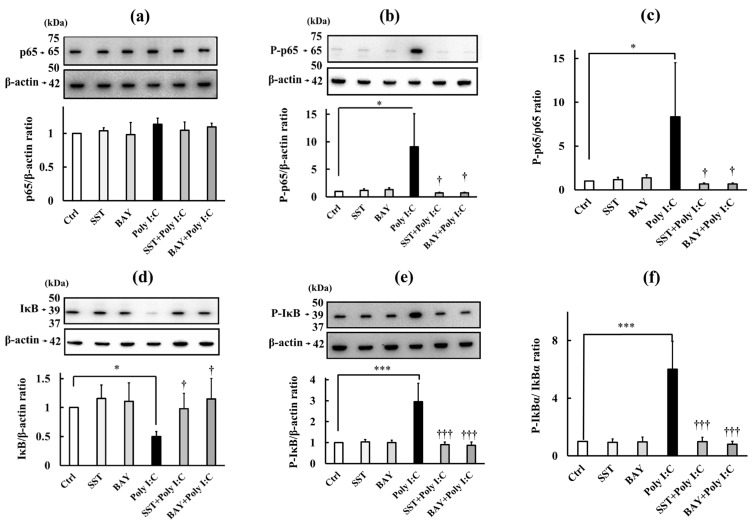
Effects of SST and BAY11-7085 on Poly I:C-induced NF-κB signaling activity. Concentrations: SST 0.3 mg/mL, BAY11-7085 (BAY) 5 μM, and Poly I:C (Poly) 10 μg/mL. (**a**,**b**) NF-κB (p65) and phosphorylated p65 (P-p65) proteins; (**c**) phosphorylation ratio (P-p65/p65); (**d**,**e**) IκBα (IκB) and phosphorylated IκBα (P-IκB) proteins; (**f**) phosphorylation ratio (P-IκB/IκB). Data are presented as mean ± SD (*n* = 3). * *p* < 0.05, *** *p* < 0.001 versus Ctrl; ^†^ *p* < 0.05, ^†††^ *p* < 0.001 versus Poly (one-way ANOVA with Dunnett’s test). The original, untrimmed Western blot images (**a**,**b**,**d**,**e**) are shown in the Supplementary Materials.

To determine whether the TJ barrier-protective effect of SST is mediated through NF-κB inhibition, we compared the effects of SST (0.3 mg/mL) with the protein levels of NF-κB (p65), IκBα (IκB), and their phosphorylated forms (P-p65 and P-IκB), as well as their phosphorylation ratios (P-p65/p65 and P-IκB/IκB), induced by Poly I:C (10 μg/mL) with those of an NF-κB inhibitor (5 μM BAY11-7085) (Figure 7).

NF-κB protein levels, measured using a p65-recognizing antibody, were not significantly altered by SST or BAY11-7085 alone, or by Poly I:C (10 μg/mL), either alone or in combination with BAY11-7085 or SST (Figure 7a), indicating that neither treatment affects total NF-κB protein abundance. Conversely, phosphorylated protein (P-p65) levels, measured using a phosphorylation-recognizing antibody, were significantly increased (*p* < 0.05) by Poly I:C (10 μg/mL). This Poly I:C-induced phosphorylation was significantly inhibited (*p* < 0.05) when SST or BAY11-7085 was co-administered, while either compound alone did not affect P-p65 protein levels (Figure 7b). Furthermore, the P-p65/p65 ratio showed that SST effectively inhibited Poly I:C-induced NF-κB phosphorylation (*p* < 0.05), comparable to the effect of the NF-κB inhibitor BAY11-7085 (Figure 7c).

IκB protein levels, measured using an IκBα-recognizing antibody, showed no significant changes with SST or BAY11-7085 alone compared with the controls. However, Poly:C (10 μg/mL) significantly reduced IκB protein levels (*p* < 0.05). This decrease was significantly inhibited (*p* < 0.05) by the combination of SST and BAY11-7085 (Figure 7d). Conversely, P-IκB levels, measured using a phosphorylation-recognizing antibody (P-IκBα), were significantly increased (*p* < 0.001) by Poly I:C (10 μg/mL). While SST or BAY11-7085 alone did not affect P-IκB protein levels, the Poly I:C-induced increase in P-IκB was significantly inhibited (*p* < 0.001) by the combination of SST or BAY11-7085 (Figure 7e). the P-IκB/IκB ratio further confirmed that SST effectively inhibited the Poly I:C-induced increase in IκB phosphorylation (*p* < 0.001), similar to the effects of the NF-κB inhibitor BAY11-7085 (Figure 7f).

These findings indicate that NF-κB signaling is closely associated with both inflammation and TJ barrier disruption, and that the TJ-protective effect of SST may be mediated, at least in part, by inhibition of this inflammatory NF-κB signaling pathway. Additional experimental data supporting these findings are presented in the Supplementary Materials (Figures S1–S24).

### 3.5. Active Components

Our results indicate that the protective effect of SST against inflammatory responses and TJ barrier disruption may be associated with suppression of NF-κB signaling pathway. This suggests that the anti-inflammatory ingredients of SST may partially contribute to these effects. To investigate this potential contribution, GL and ILQG were selected as candidate ingredients based on their known anti-inflammatory activity [36] and pharmacokinetic profiles [22,37,38]. Notably, previous studies have reported that GL and ILQG exert protective effects against epithelial barrier dysfunction in various tissues [39,40], further supporting their involvement in the efficacy of SST.

Regarding their pharmacokinetics, GL is a triterpenoid glycoside, whereas ILQG is the aglycone of the flavonoid glycosides isoliquiritin apioside and isoliquiritin. Orally administered GL is metabolized by intestinal bacteria into the aglycone GA, which retains equivalent biological activity and is absorbed through the intestinal tract. Similarly, ILQG is absorbed presystemically following bacterial conversion from isoliquiritin apioside and isoliquiritin [22,37,38]. Consequently, as preliminary investigation for future detailed ingredient studies, this study focused on these two anti-inflammatory ingredients as candidates, which are known to be reliably absorbed into the bloodstream following the oral administration of SST.

**Figure 8:**
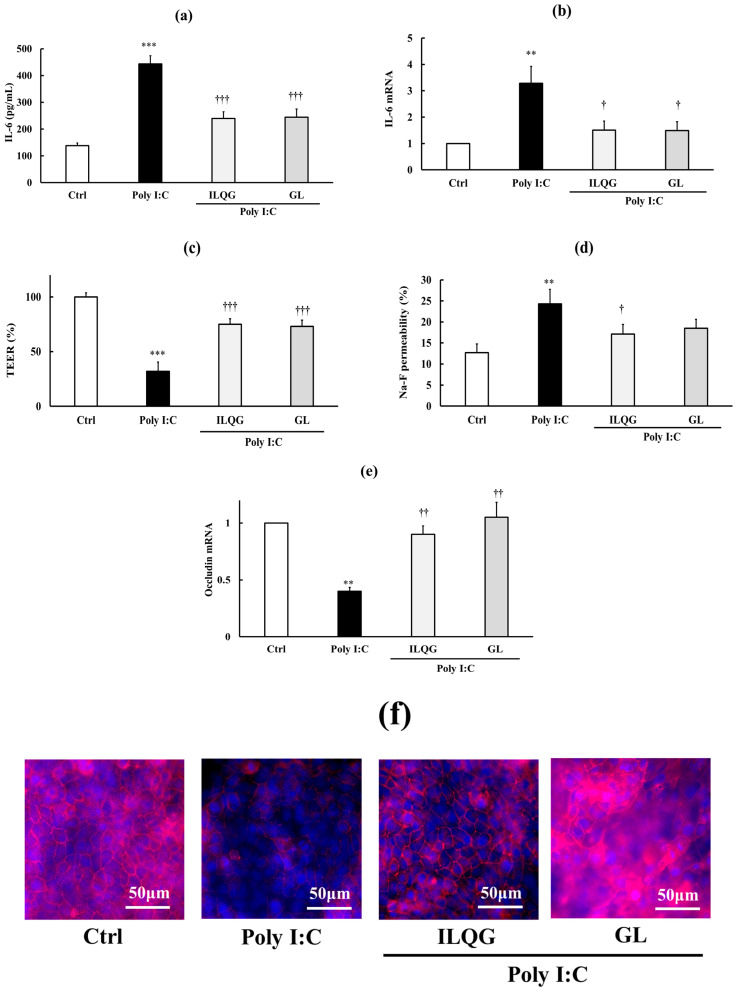
Effects of GL and ILQG on Poly I:C-induced inflammation and TJ barrier disruption. (**a**,**b**) Inflammatory markers: IL-6 medium concentration and IL-6 mRNA expression. (**c**–**f**) TJ barrier markers: TEER, Na–F permeability, occludin mRNA expression, and immunofluorescent-stained protein. Occludin is shown in red and nuclei are stained with DAPI (blue). Data are presented as the mean ± SD (*n* = 3). ** *p* < 0.01, *** *p* < 0.001 vs. Ctrl; ^†^ *p* < 0.05, ^††^ *p* < 0.01, ^†††^ *p* < 0.001 vs. Poly (one-way ANOVA with Dunnett’s test).

Figure 8 shows the effects of ILQG and GA on Poly I:C-induced inflammation, TJ barrier disruption, and NF-κB signaling activity. Both components are known to exert anti-inflammatory and neuroprotective effects [36,41]. In this study, ILQG and GA were applied at 25 µM, a concentration determined in preliminary experiments to prevent TJ barrier disruption and increase inflammatory mediators across multiple inflammation models.

**Figure 9:**
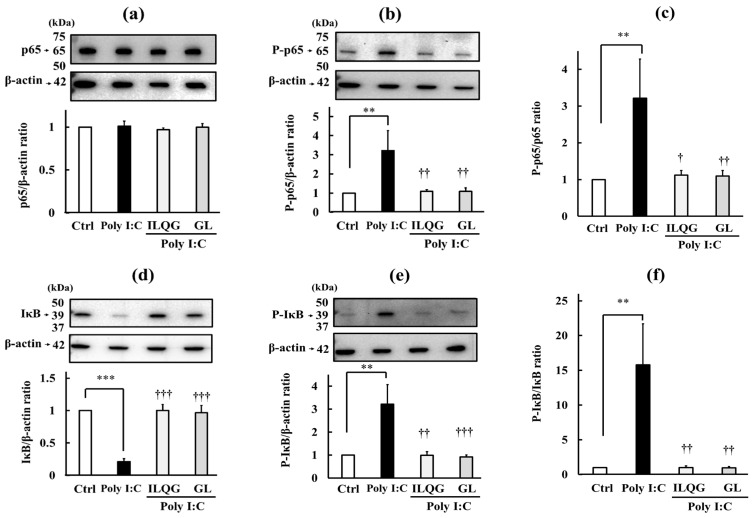
Effects of GL and ILQG on Poly I:C-induced NF-κB signaling activity. Concentrations: SST 0.3 mg/mL, Poly 10 μg/mL, ILQG 25 µM, and GL 25 µM. (**a**,**b**) NF-κB (p65) and P-p65; (**c**) P-p65/p65; (**d**,**e**) IκB and P-IκB; (**f**) P-IκB/IκB. Data are presented as mean ± SD (*n* = 3). ** *p* < 0.01, *** *p* < 0.001 versus Ctrl; ^†^ *p* < 0.05, ^††^ *p* < 0.01, ^†††^ *p* < 0.001 versus Poly (one-way ANOVA with Dunnett’s test). The original, untrimmed Western blot images (**a**,**b**,**d**,**e**) are shown in the Supplementary Materials.

Treatment with ILQG or GA significantly ameliorated Poly I:C-induced inflammation (*p* < 0.01–0.001), as evidenced by the significantly decreased IL-6 concentration in the culture medium and reduced IL-6 mRNA expression (*p* < 0.05–0.001), and protected against TJ barrier disruption, reflected by increased TEER (*p* < 0.001), decreased Na–F permeability (*p* < 0.01), and increased occludin mRNA expression (*p* < 0.01) and protein immunostaining (Figure 8a–f). Furthermore, both components significantly inhibited (*p* < 0.01) the increased phosphorylation of NF-κB p65 protein (*p* < 0.01) (Figure 9a–c) and IκB protein (*p* < 0.001) (Figure 9d–f), indicating that both components significantly inhibited Poly I:C-induced NF-κB signaling. These protective effects against inflammation, TJ barrier disruption, and NF-κB signaling activation were completely similar to those observed with SST (Figure 5a–f, Figure 6a–f and Figure 7a–f). Overall, we cannot definitively conclude that GL and ILQG are the main active ingredients of SST; however, these results suggest that anti-inflammatory ingredients such as GL and ILQG may contribute, in part, to its effects.

### 3.6. Limitations of the Study

This study has several limitations. First, the specific target site of SST within the NF-κB signaling pathway has not yet been fully elucidated. The protective effects of SST on IL-6 production induced by pro-inflammatory stimuli (LPS, H_2_O_2_, TNF-α, and Poly I:C) and TJ barrier disruption were similar to those observed with NF-κB inhibitors that suppress IKK activity and IκB phosphorylation, suggesting that SST may exert its effects, at least in part, through modulation of the NF-κB signaling pathway. However, we cannot definitively conclude that SST directly targets IKK or IκB. This is because the NF-κB signaling pathway is closely interconnected with other signaling cascades, such as the MAPK and STAT3 pathways, which are involved in regulating inflammatory responses and other cellular processes [16]. For instance, the activation of the MAPK activates the AP-1 transcription factor to synergistically promote the expression of proinflammatory cytokines, and its components, such as c-Jun N-terminal kinase (JNK) and p38 mitogen-activated protein kinase (p38), are known to trigger IκB degradation, thereby activating NF-κB [16,42,43]. Furthermore, the STAT pathway (particularly STAT3), often activated by cytokines via the JAK/STAT axis, can cooperate with NF-κB to regulate genes involved in both the inflammatory response and TJ protein synthesis [39]. Therefore, further studies are needed to delineate the mechanisms by which SST inhibits NF-κB signaling, including its direct effects on IKK and IκB, as well as its potential indirect effects through the MAPK and STAT3 pathways. Additionally, as NF-κB activation was primarily assessed through the phosphorylation of p65 and IκBα, further studies into its nuclear translocation and transcriptional activity would provide a more comprehensive understanding.

In the present study, SST consistently demonstrated anti-inflammatory and TJ protective effects in all four inflammation provocation models. Therefore, the Poly I:C model was selected to investigate the mechanism of action using NF-κB inhibitors. While we recognize the importance of examining the NF-κB pathway in all models, the consistent protective trend observed in this study suggests that the findings from the Poly I:C model may reflect a common mechanism underlying the pharmacological action of SST.

Although IL-6 was used as a representative inflammatory marker, the present study does not establish a direct causal relationship between IL-6 production and TJ barrier disruption. Both processes may be regulated by NF-κB signaling but could occur through partially independent pathways. Hence, it remains unclear whether the protective effects of SST are mediated directly through NF-κB inhibition or are secondary to the suppression of cytokines like IL-6. Further investigations using IL-6-neutralizing antibodies or similar approaches are required to decouple these two processes and clarify the precise underlying mechanism. In addition, as this study focused solely on IL-6 as a representative marker, other NF-κB-regulated cytokines were not assessed. Consequently, the broader inflammatory profile associated with SST treatment is not yet fully understood, and further research is warranted to delineate these effects.

Second, although this study suggests that anti-inflammatory components such as ILQG and GL may contribute, in part, to the effects of SST, its full range of active components remains unknown. SST comprises eight crude drugs and has been reported to contain at least 174 bioactive components [44], including additional anti-inflammatory agents, such as flavonoids from *Glycyrrhiza* (liquiritin apioside, liquiritin, liquiritigenin) [37], alkaloids from *Ephedra* herb (ephedrine, pseudoephedrine) [45], phenols from processed ginger (6-shogaol, 6-gingerol) [46], phenylpropanoids from cinnamon bark (cinnamic acid, cinnamic aldehyde) [47], lignans from *Schisandra* fruit (schizandrin, gomisin) [48], and monoterpenoids from peony root (paeoniflorin, albiflorin) [49]. Consequently, further investigation into the effects of these other components is warranted.

Furthermore, for oral SST formulations to exert pharmacological effects in vivo, the active components must be absorbed into the bloodstream from the intestinal tract [50]. Therefore, elucidating the in vivo pharmacokinetics of SST components is essential. In interpreting our in vitro findings, clinical feasibility and pharmacokinetic profiles must be carefully considered. To bridge this gap, we focused on GL and ILQG as representative anti-inflammatory markers, as their systemic absorption has been well-documented in pharmacokinetic studies of other Kampo medicines, such as yokukansan [50] and shakuyakukanzoto [22]. To our knowledge, no pharmacokinetic studies of SST have been reported to date. Until such data become available, it is advisable to focus on components that are detectable in the bloodstream—as exemplified by ILQG and GL in this study—guided by existing pharmacokinetic data from individual crude drugs or related Kampo medicines.

However, we recognize that the concentrations used with the in vitro model may not directly correspond to the peak plasma concentrations (Cmax) achievable in clinical practice. The pharmacological efficacy of Kampo medicines is often attributed not to a single high-dose component, but rather the additive and synergistic effects of multiple low-concentration ingredients [50]. Thus, while our results provide a mechanistic insight into the direct protective effect of SST on epithelial cells, further in vivo studies using animal models and clinical pharmacokinetic monitoring are essential to validate these findings. The accumulation of these future investigations will clarify the complex interactions between blood-detected components and the actual clinical impact on mucosal integrity.

Third, TJ integrity was primarily assessed by occludin expression. Since TJs comprise various proteins, such as claudins and ZO-1, future research should incorporate additional components to fully characterize SST-mediated barrier regulation.

Finally, because this study was conducted using an in vitro epithelial cell model, the results may not fully reflect the complex in vivo environment of the airway epithelium. Thus, further in vivo investigations are needed to establish clinical relevance. House dust mite (HDM)-induced asthmatic mice are widely used to study inflammatory airway diseases such as allergic rhinitis and asthma [49,51]. SST has also been shown in these models to ameliorate inflammatory symptoms through the inhibition of NF-κB activation [52,53]. Furthermore, a recent study reported disruption of epithelial TJ barrier function in HDM-induced mice [54,55], suggesting that this model is suitable for the in vivo validation of our in vitro results. Future studies should utilize this model to verify the protective role of SST in maintaining TJ integrity.

## 4. Conclusions

This study suggests that (1) activation of the NF-κB signaling pathway might be associated with both inflammatory responses and TJ barrier disruption; (2) SST could reduce these effects, potentially through modulation of NF-κB signaling; and (3) ILQG and GL may contribute in part to these activities. This study provides the first evidence suggesting that SST may exert anti-inflammatory and epithelial barrier-protective effects, possibly via the suppression of the NF-κB signaling pathway.

## Data Availability

The data presented in this study are available from the corresponding author upon reasonable request.

## Supplementary Materials

The following supporting information can be downloaded at: https://www.mdpi.com/article/10.3390/biology15080603/s1. Figure S1. p65 (Replicate 1); Figure S2. p65 (Replicate 2); Figure S3. p65 (Replicate 3); Figure S4. P-p65 (Replicate 1); Figure S5. P-p65 (Replicate 2); Figure S6. P-p65 (Replicate 3); Figure S7. IκBα (Replicate 1); Figure S8. IκBα (Replicate 2); Figure S9. IκBα (Replicate 3); Figure S10. P-IκBα (Replicate 1); Figure S11. P-IκBα (Replicate 2); Figure S12. P-IκBα (Replicate 3); Figure S13. p65 (Replicate 1); Figure S14. p65 (Replicate 2); Figure S15. p65 (Replicate 3); Figure S16. P-p65 (Replicate 1); Figure S17. P-p65 (Replicate 2); Figure S18. P-p65 (Replicate 3); Figure S19. IκBα (Replicate 1); Figure S20. IκBα (Replicate 2); Figure S21. IκBα (Replicate 3); Figure S22. P-IκBα (Replicate 1); Figure S23. P-IκBα (Replicate 2); Figure S24. P-IκBα (Replicate 3).

## Abbreviations

The following abbreviations are used in this manuscript:

16HBE	Human bronchial epithelial
GL	Glycyrrhizin
IκB	Inhibitor of κB
IKK	IκB kinase
IL	Interleukin
ILQG	Isoliquiritigenin
LPS	Lipopolysaccharide
MAPK	Mitogen-activated protein kinase
AP-1	Activator protein-1
JAK	Janus kinase
STAT	Signal transducer and activator of transcription
Na–F	Sodium fluorescein
NF-κB	Nuclear factor kappa B
P-p65	Phosphorylated p65
P-IκB	Phosphorylated IκB
Poly I:C	Polyinosinic–polycytidylic acid
SD	Standard Deviation
SST	*Shoseiryuto*
TEER	Transepithelial electrical resistance
TNF-α	Tumor necrosis factor-α
TJ	Tight junction
XQLT	Xiao-Qing-Long-Tang
