# Supplementary material for: Shoseiryuto May Prevent Bronchial Epithelial Tight Junction Disruption by Inhibiting the Inflammatory NF-κB Signaling Pathway
Source: Biology (Basel). 2026 Apr 11;15(8):603. doi: 10.3390/biology15080603 (PMC13113397; doi:10.3390/biology15080603)
Supplement: Supplementary file 1 [file biology-15-00603-s001.zip › biology-4187309-supplementary.pdf]

Supplementary Figure S1– p65 (Replicate 1)

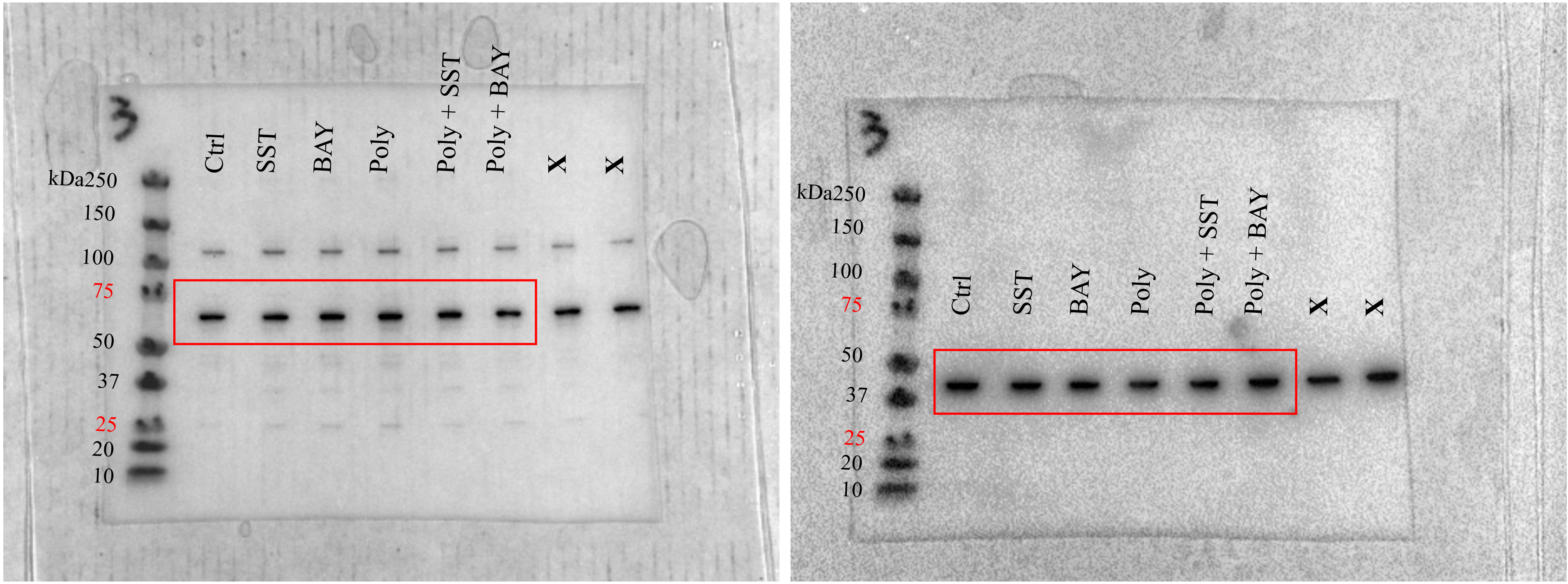

Lane assignment (from left to right):
Lane 1: Molecular weight marker
Lane 2: Empty lane
Lane 3: Control
Lane 4: Empty lane
Lane 5: SST
Lane 6: Empty lane
Lane 7: BAY11-7085
Lane 8: Empty lane
Lane 9: Poly I:C
Lane 10: Empty lane
Lane 11: Poly I:C + SST
Lane 12: Empty lane
Lane 13: Poly I:C + BAY11-7085
Lane 14–17: Unused lanes

Supplementary Figure S1.
Uncropped Western blot corresponding to Figure 7 (a), (p65), Replicate 1.Target protein and β-actin were detected on the same membrane using the same protein lysates. The images show the full membranes used to generate the main figure. Red boxes indicate the cropped regions presented in the manuscript. All lanes are shown, including unused lanes. No brightness or contrast adjustments were applied to individual bands.

Supplementary Figure S2– p65 (Replicate 2)

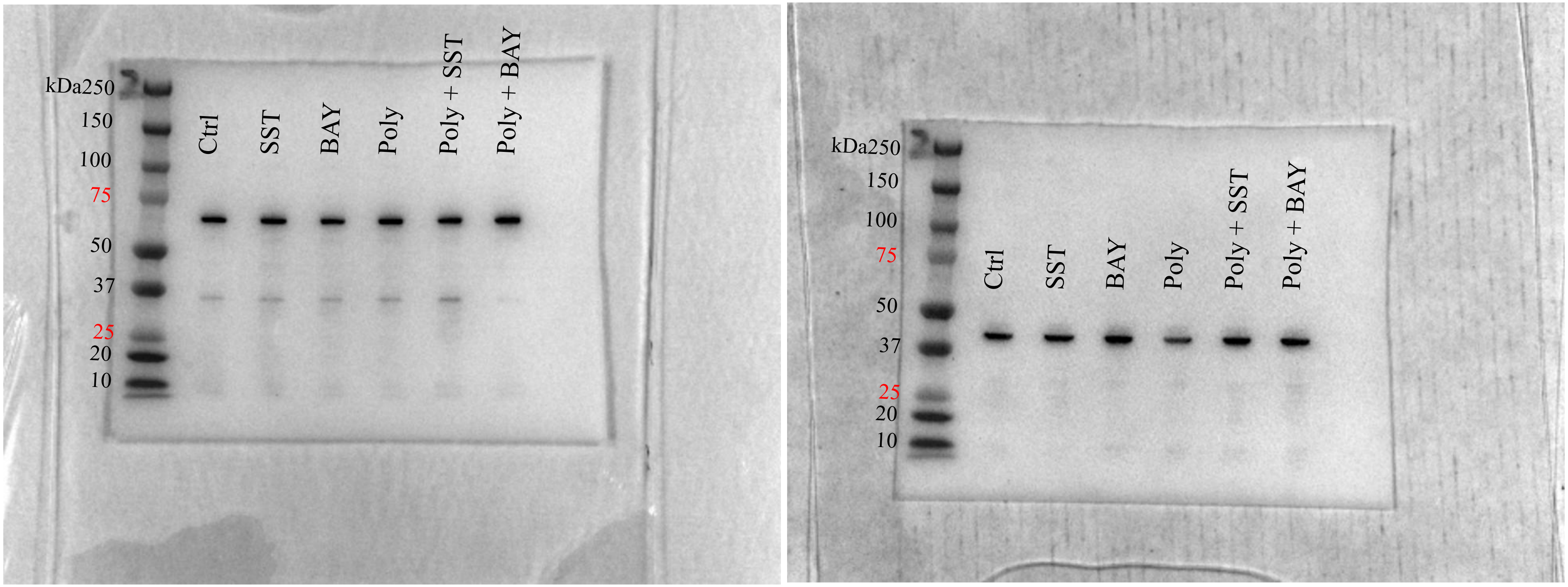

Lane assignment (from left to right):
Lane 1: Molecular weight marker
Lane 2: Empty lane
Lane 3: Control
Lane 4: Empty lane
Lane 5: SST
Lane 6: Empty lane
Lane 7: BAY11-7085
Lane 8: Empty lane
Lane 9: Poly I:C
Lane 10: Empty lane
Lane 11: Poly I:C + SST
Lane 12: Empty lane
Lane 13: Poly I:C + BAY11-7085
Lane 14–17: Empty lane

Supplementary Figure S2.
Uncropped Western blot corresponding to Figure 7 (a) (p65), Replicate 2. Target protein and β-actin were detected on the same membrane using the same protein lysates. The images show the full membranes from an independent biological replicate used for quantitative densitometric analysis presented in Figure 7 (a). All lanes are shown, including unused lanes. No brightness or contrast adjustments were applied to individual bands.

Supplementary Figure S3– p65 (Replicate 3)

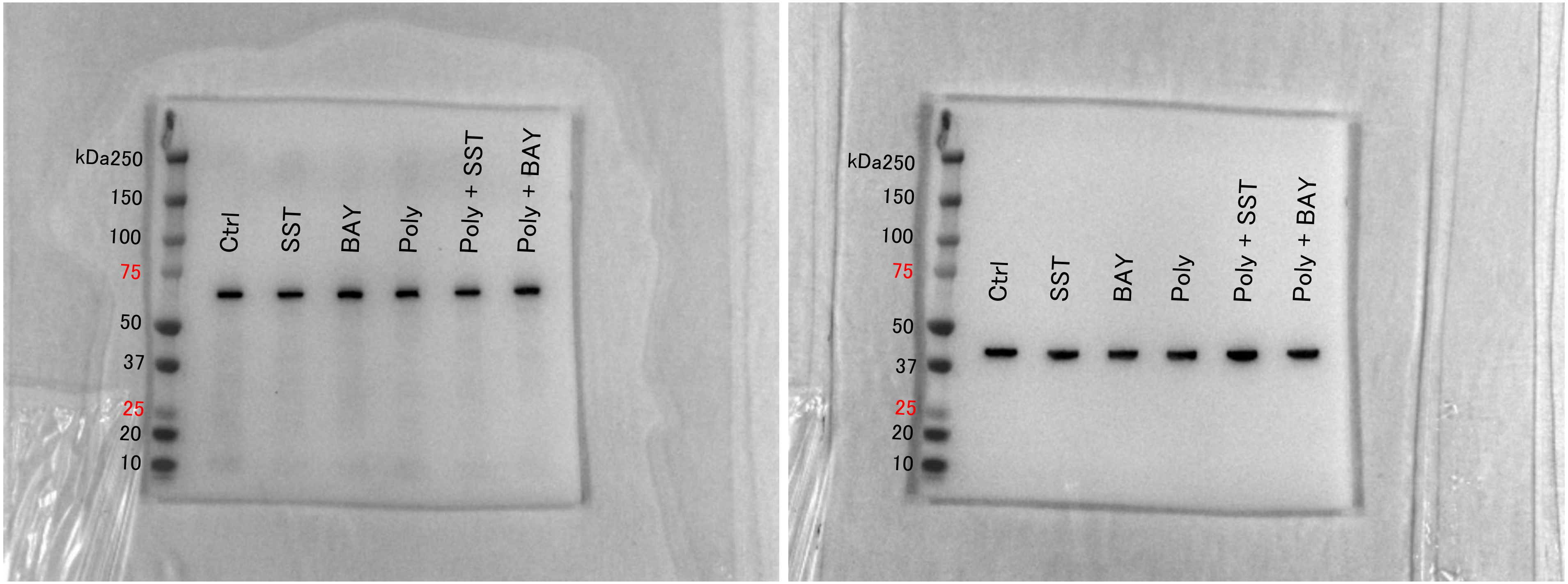

Lane assignment (from left to right):
Lane 1: Molecular weight marker
Lane 2: Empty lane
Lane 3: Control
Lane 4: Empty lane
Lane 5: SST
Lane 6: Empty lane
Lane 7: BAY11-7085
Lane 8: Empty lane
Lane 9: Poly I:C
Lane 10: Empty lane
Lane 11: Poly I:C + SST
Lane 12: Empty lane
Lane 13: Poly I:C + BAY11-7085
Lane 14–17: Empty lane

Supplementary Figure S3.
Uncropped Western blot corresponding to Figure 7 (a) (p65), Replicate 3. Target protein and β-actin were detected on the same membrane using the same protein lysates. The images show the full membranes from an independent biological replicate used for quantitative densitometric analysis presented in Figure 7 (a). All lanes are shown, including unused lanes. No brightness or contrast adjustments were applied to individual bands.

Supplementary Figure S4– P-p65 (Replicate 1)

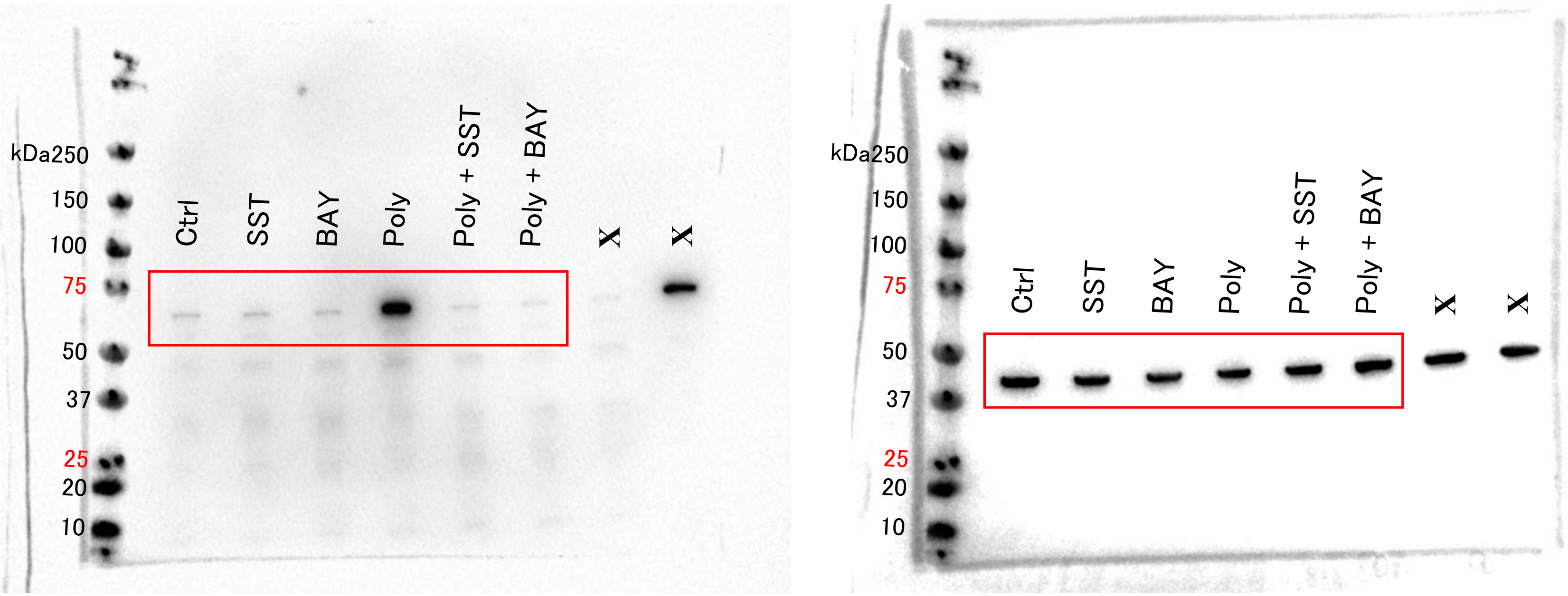

Lane assignment (from left to right):
Lane 1: Molecular weight marker
Lane 2: Empty lane
Lane 3: Control
Lane 4: Empty lane
Lane 5: SST
Lane 6: Empty lane
Lane 7: BAY11-7085
Lane 8: Empty lane
Lane 9: Poly I:C
Lane 10: Empty lane
Lane 11: Poly I:C + SST
Lane 12: Empty lane
Lane 13: Poly I:C + BAY11-7085
Lane 14–17: Unused lanes

Supplementary Figure S4.
Uncropped Western blot corresponding to Figure 7 (b), (P-p65), Replicate 1.Target protein and β-actin were detected on the same membrane using the same protein lysates. The images show the full membranes used to generate the main figure. Red boxes indicate the cropped regions presented in the manuscript. All lanes are shown, including unused lanes. No brightness or contrast adjustments were applied to individual bands.

Supplementary Figure S5– P-p65 (Replicate 2)

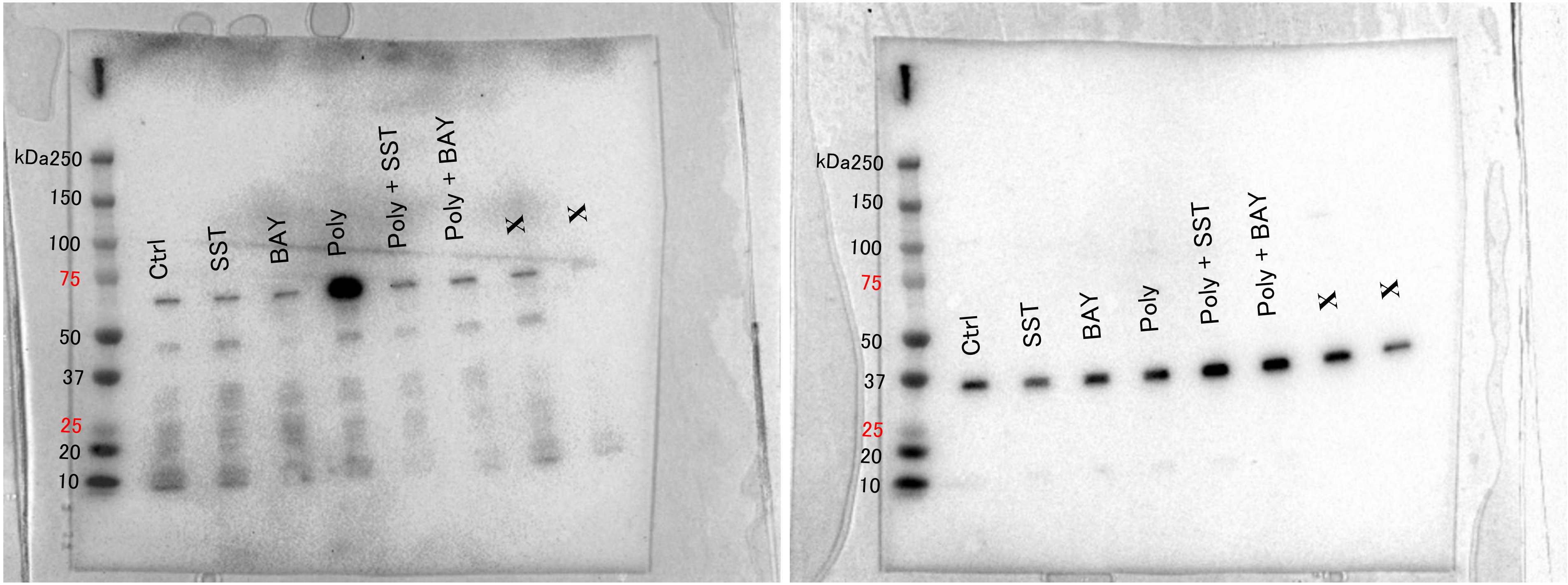

Lane assignment (from left to right):
Lane 1: Molecular weight marker
Lane 2: Empty lane
Lane 3: Control
Lane 4: Empty lane
Lane 5: SST
Lane 6: Empty lane
Lane 7: BAY11-7085
Lane 8: Empty lane
Lane 9: Poly I:C
Lane 10: Empty lane
Lane 11: Poly I:C + SST
Lane 12: Empty lane
Lane 13: Poly I:C + BAY11-7085
Lane 14–17: Unused lanes

Supplementary Figure S5.
Uncropped Western blot corresponding to Figure 7 (b), (P-p65), Replicate 2. Target protein and β-actin were detected on the same membrane using the same protein lysates. The images show the full membranes from an independent biological replicate used for quantitative densitometric analysis presented in Figure 7 (b). All lanes are shown, including unused lanes. No brightness or contrast adjustments were applied to individual bands.

Supplementary Figure S6– P-p65 (Replicate 3)

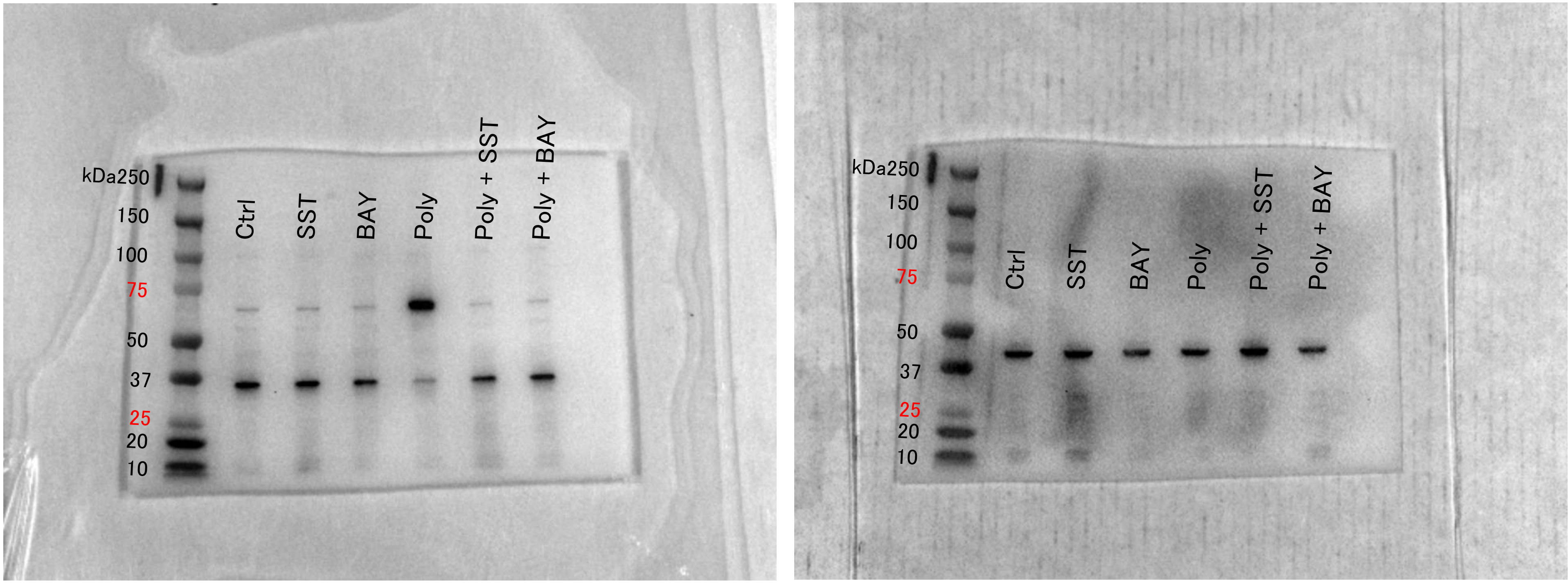

Lane assignment (from left to right):
Lane 1: Molecular weight marker
Lane 2: Empty lane
Lane 3: Control
Lane 4: Empty lane
Lane 5: SST
Lane 6: Empty lane
Lane 7: BAY11-7085
Lane 8: Empty lane
Lane 9: Poly I:C
Lane 10: Empty lane
Lane 11: Poly I:C + SST
Lane 12: Empty lane
Lane 13: Poly I:C + BAY11-7085
Lane 14–17: Empty lane

Supplementary Figure S6.
Uncropped Western blot corresponding to Figure 7 (b) (P-p65) and Figure 7 (d) (IκBα), Replicate 3. P-p65, IκBα and β-actin were detected in simultaneously processed immunoblots using the same protein lysates from an independent biological replicate. The same immunoblots were used for quantitative densitometric analyses presented in Figures 7 (b) and 7 (d). All lanes are shown, including unused lanes. No brightness or contrast adjustments were applied to individual bands.

Supplementary Figure S7– IκBα (Replicate 1)

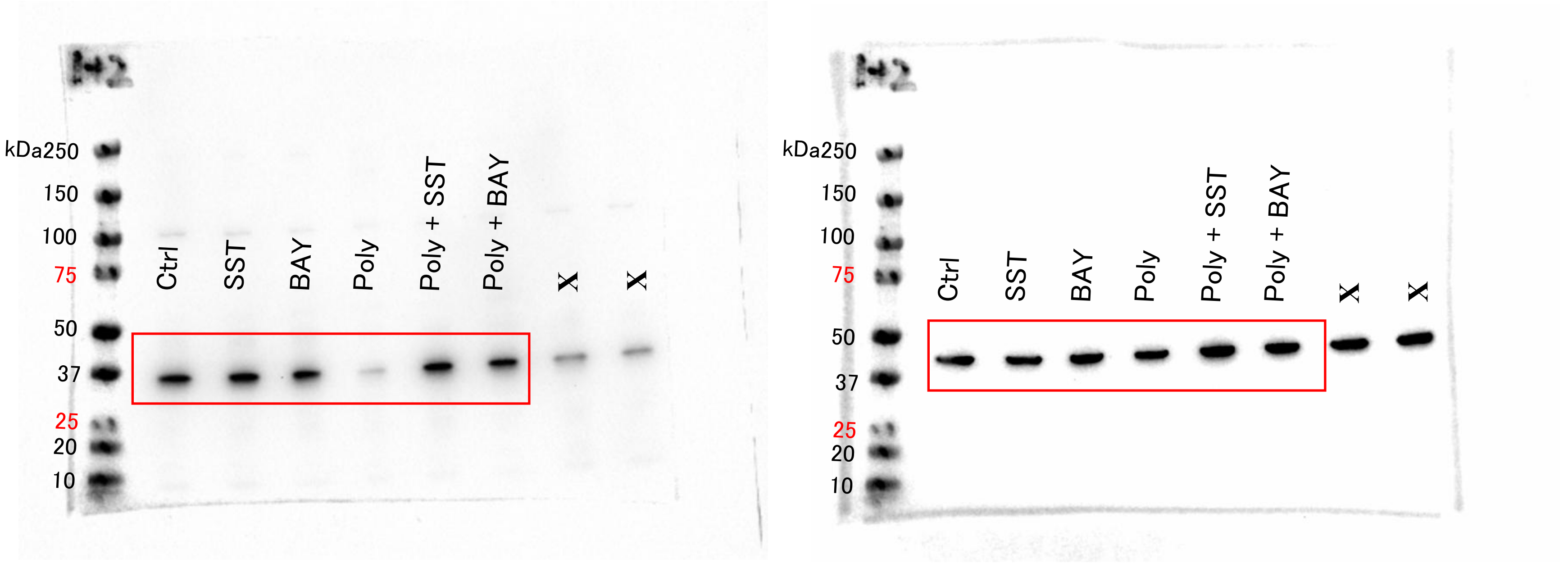

Lane assignment (from left to right):
Lane 1: Molecular weight marker
Lane 2: Empty lane
Lane 3: Control
Lane 4: Empty lane
Lane 5: SST
Lane 6: Empty lane
Lane 7: BAY11-7085
Lane 8: Empty lane
Lane 9: Poly I:C
Lane 10: Empty lane
Lane 11: Poly I:C + SST
Lane 12: Empty lane
Lane 13: Poly I:C + BAY11-7085
Lane 14–17: Unused lanes

Supplementary Figure S7.
Uncropped Western blot corresponding to Figure 7 (d), (IκBα), Replicate 1.Target protein and β-actin were detected on the same membrane using the same protein lysates. The images show the full membranes used to generate the main figure. Red boxes indicate the cropped regions presented in the manuscript. All lanes are shown, including unused lanes. No brightness or contrast adjustments were applied to individual bands.

Supplementary Figure S8– IκBα (Replicate 2)

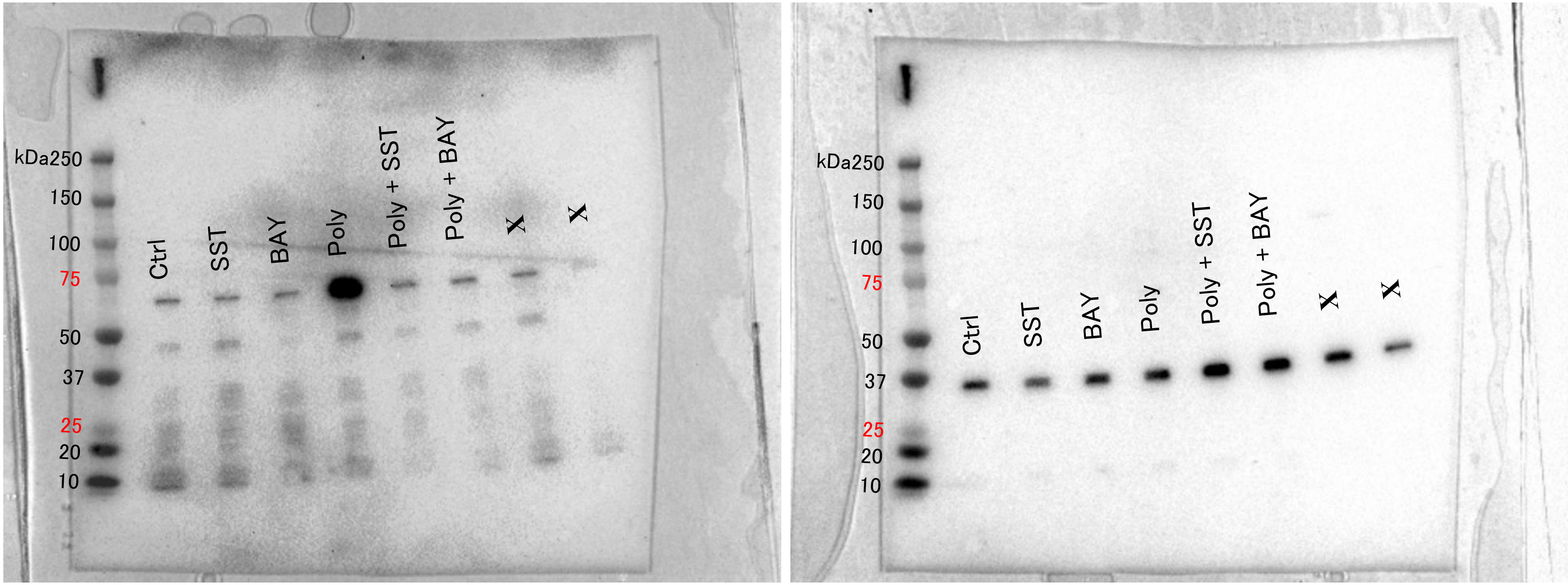

Lane assignment (from left to right):
Lane 1: Molecular weight marker
Lane 2: Empty lane
Lane 3: Control
Lane 4: Empty lane
Lane 5: SST
Lane 6: Empty lane
Lane 7: BAY11-7085
Lane 8: Empty lane
Lane 9: Poly I:C
Lane 10: Empty lane
Lane 11: Poly I:C + SST
Lane 12: Empty lane
Lane 13: Poly I:C + BAY11-7085
Lane 14–17: Unused lanes

Supplementary Figure S8.
Uncropped Western blot corresponding to Figure 7 (d), (IκBα), Replicate 2. Target protein and β-actin were detected on the same membrane using the same protein lysates. The images show the full membranes from an independent biological replicate used for quantitative densitometric analysis presented in Figure 7 (d). All lanes are shown, including unused lanes. No brightness or contrast adjustments were applied to individual bands.

Supplementary Figure S9– IκBα (Replicate 3)

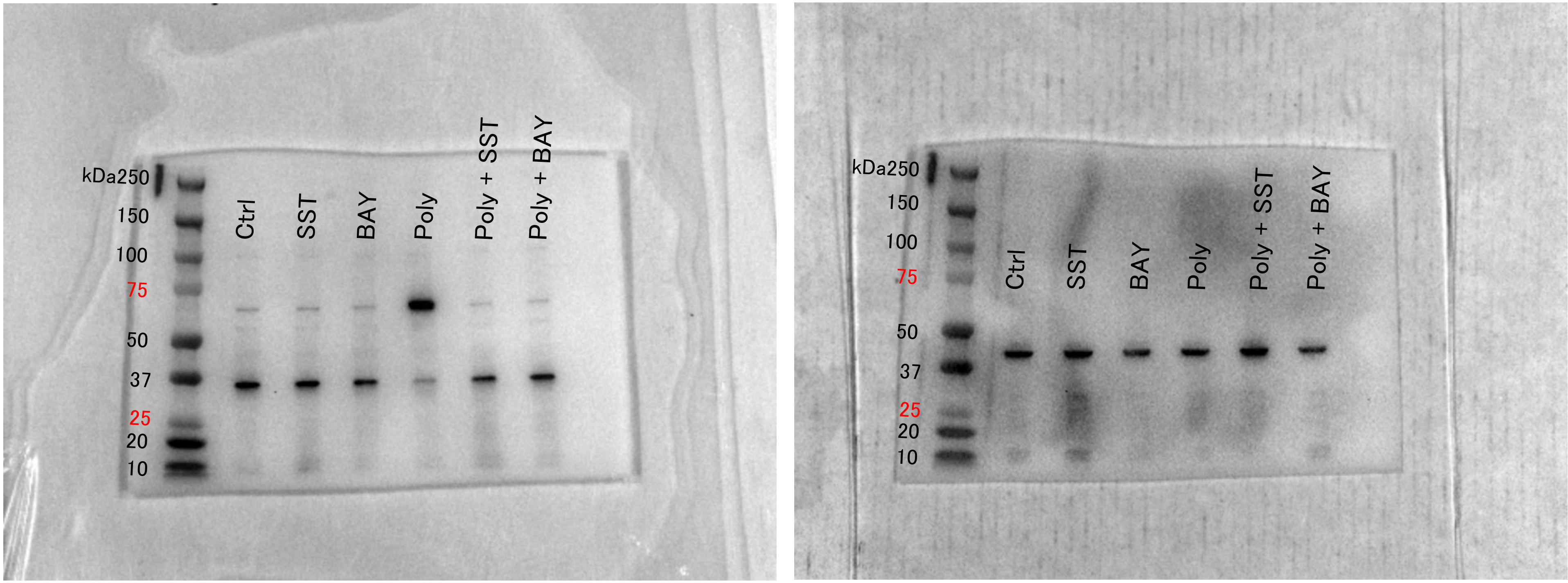

Lane assignment (from left to right):
Lane 1: Molecular weight marker
Lane 2: Empty lane
Lane 3: Control
Lane 4: Empty lane
Lane 5: SST
Lane 6: Empty lane
Lane 7: BAY11-7085
Lane 8: Empty lane
Lane 9: Poly I:C
Lane 10: Empty lane
Lane 11: Poly I:C + SST
Lane 12: Empty lane
Lane 13: Poly I:C + BAY11-7085
Lane 14–17: Empty lane

Supplementary Figure S9.
Uncropped Western blot corresponding to Figure 7 (d) (IκBα), Replicate 3. This membrane is the same as that shown in Supplementary Figure S6.IκBα and β-actin signals from the same immunoblot were used forquantitative analysis in Figure 7 (d).

Supplementary Figure S10–P- IκBα (Replicate 1)

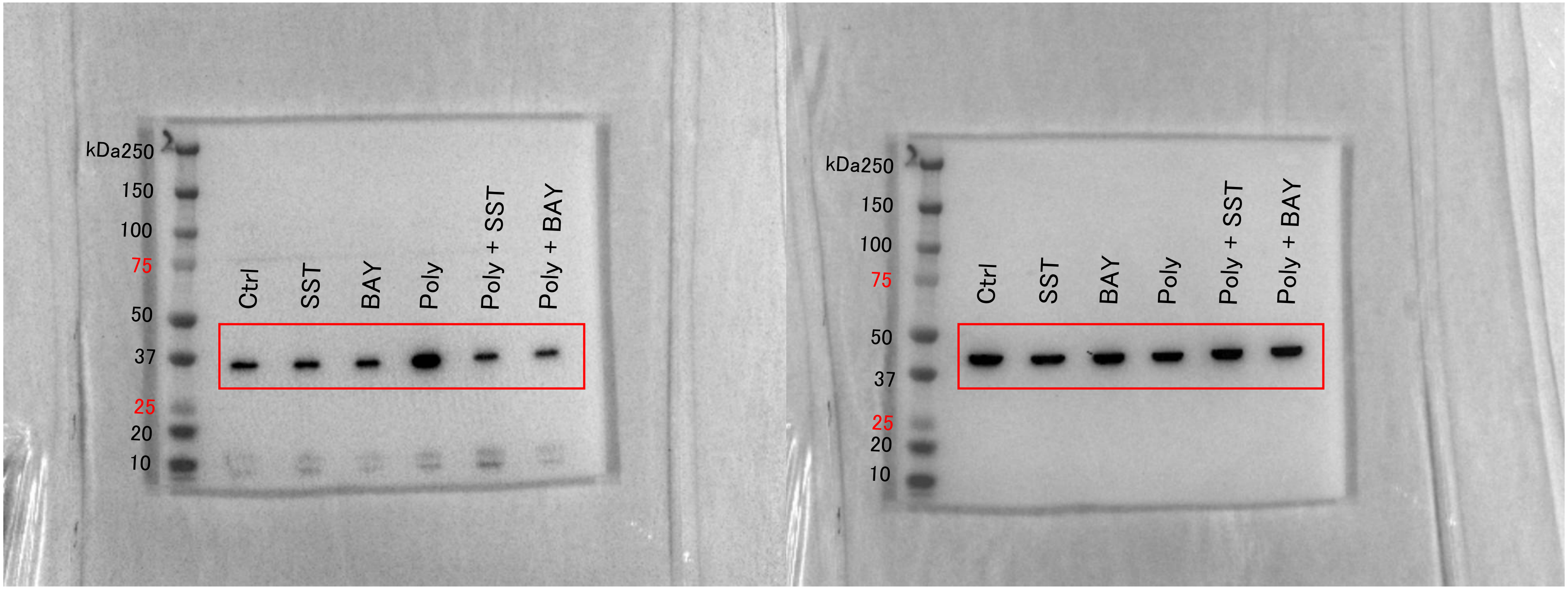

Lane assignment (from left to right):
Lane 1: Molecular weight marker
Lane 2: Empty lane
Lane 3: Control
Lane 4: Empty lane
Lane 5: SST
Lane 6: Empty lane
Lane 7: BAY11-7085
Lane 8: Empty lane
Lane 9: Poly I:C
Lane 10: Empty lane
Lane 11: Poly I:C + SST
Lane 12: Empty lane
Lane 13: Poly I:C + BAY11-7085
Lane 14–17: Empty lane

Supplementary Figure S10.
Uncropped Western blot corresponding to Figure 7 (e), (P-IκBα), Replicate 1.Target protein and β-actin were detected on the same membrane using the same protein lysates. The images show the full membranes used to generate the main figure. Red boxes indicate the cropped regions presented in the manuscript. All lanes are shown, including unused lanes. No brightness or contrast adjustments were applied to individual bands.

Supplementary Figure S11–P- IκBα (Replicate 2)

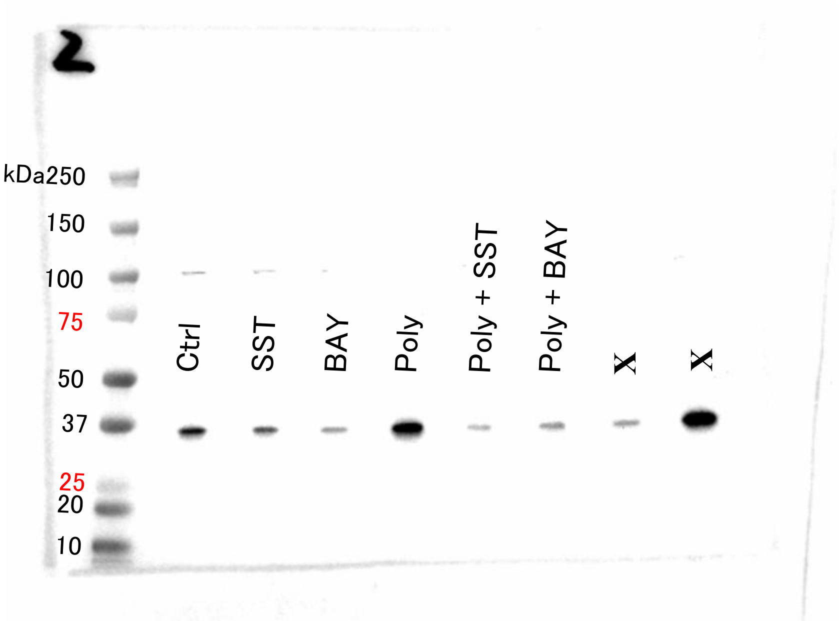

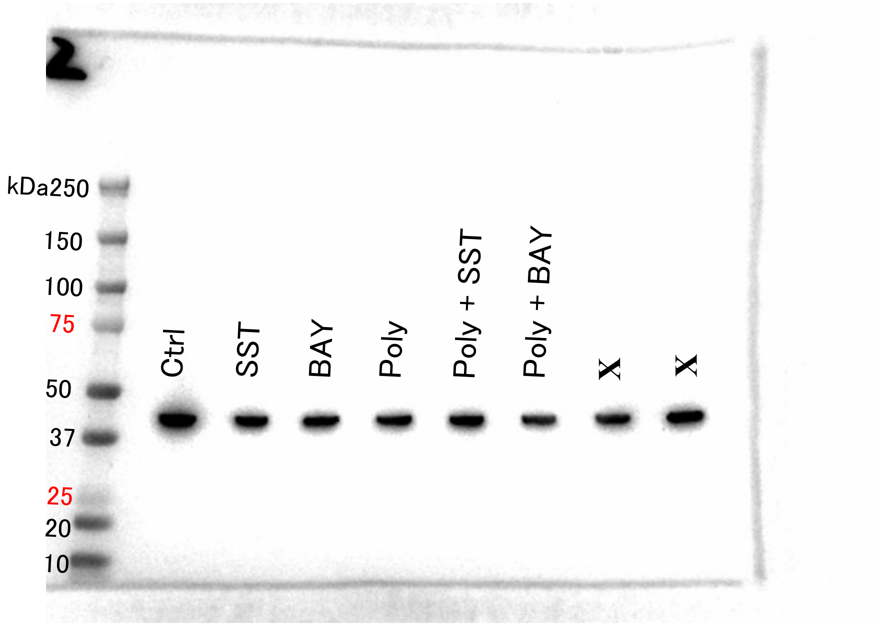

Lane assignment (from left to right):
Lane 1: Molecular weight marker
Lane 2: Empty lane
Lane 3: Control
Lane 4: Empty lane
Lane 5: SST
Lane 6: Empty lane
Lane 7: BAY11-7085
Lane 8: Empty lane
Lane 9: Poly I:C
Lane 10: Empty lane
Lane 11: Poly I:C + SST
Lane 12: Empty lane
Lane 13: Poly I:C + BAY11-7085
Lane 14–17: Unused lanes

Supplementary Figure S11.
Uncropped Western blot corresponding to Figure 7 (e), (P-IκBα), Replicate 2. P-IκBα and β-actin were detected on the same membrane using the same protein lysates. The images show the full membranes from an independent biological replicate used for quantitative densitometric analysis presented in Figure 7 (e). All lanes are shown, including unused lanes. No brightness or contrast adjustments were applied to individual bands.

Supplementary Figure S12– P-IκBα (Replicate 3)

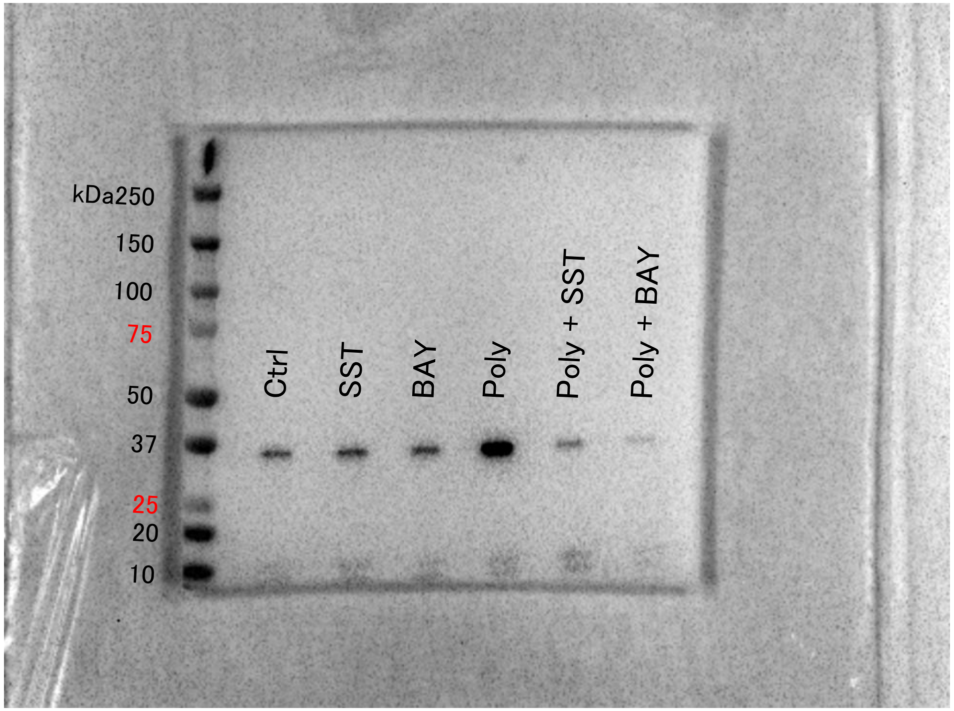

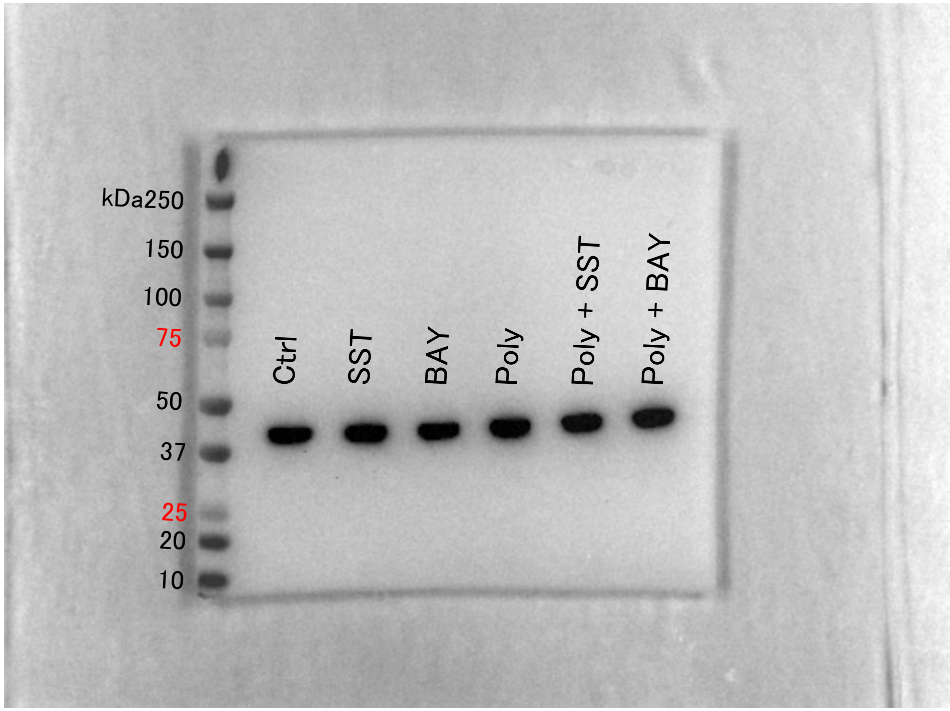

Lane assignment (from left to right):
Lane 1: Molecular weight marker
Lane 2: Empty lane
Lane 3: Control
Lane 4: Empty lane
Lane 5: SST
Lane 6: Empty lane
Lane 7: BAY11-7085
Lane 8: Empty lane
Lane 9: Poly I:C
Lane 10: Empty lane
Lane 11: Poly I:C + SST
Lane 12: Empty lane
Lane 13: Poly I:C + BAY11-7085
Lane 14–17: Empty lane

Supplementary Figure S12.
Uncropped Western blot corresponding to Figure 7 (e) (P-IκBα), Replicate 3. P-IκBα and β-actin were detected in simultaneously processed immunoblots using the same protein lysates from an independent biological replicate. The same immunoblots were used for quantitative densitometric analyses presented in Figures 7 (e) . All lanes are shown, including unused lanes. No brightness or contrast adjustments were applied to individual bands.

Supplementary Figure S13– p65 (Replicate 1)

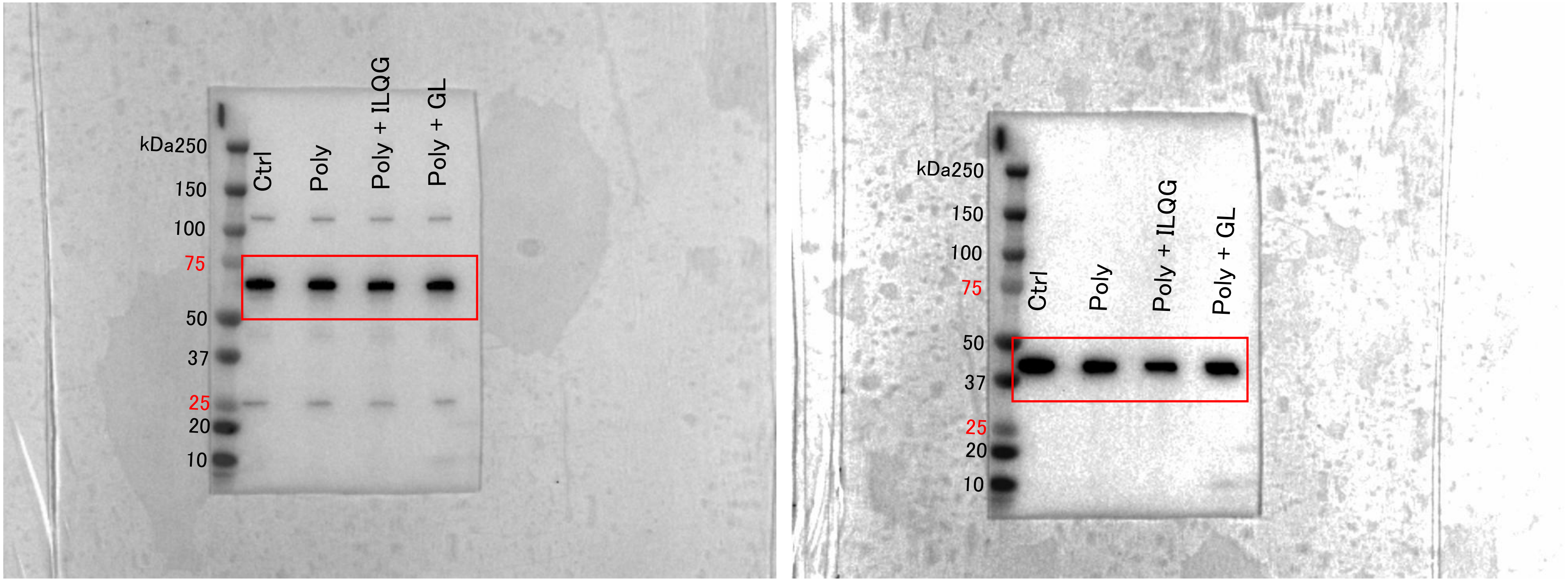

Lane assignment (from left to right):

Lane 1: Molecular weight marker
Lane 2: Control
Lane 3: Empty lane
Lane 4: Poly I:C
Lane 5: Empty lane
Lane 6: Poly I:C +ILQG
Lane 7: Empty lane
Lane 8: Poly I:C +GL

Supplementary Figure S13.
Uncropped Western blot corresponding to Figure 9 (a)(p65), Replicate 1.Target protein and β-actin were detected on the same membrane using the same protein lysates. The images show the full membranes used to generate the main figure. Red boxes indicate the cropped regions presented in the manuscript. All lanes are shown, including unused lanes. No brightness or contrast adjustments were applied to individual bands.

Supplementary Figure S14– p65 (Replicate 2)

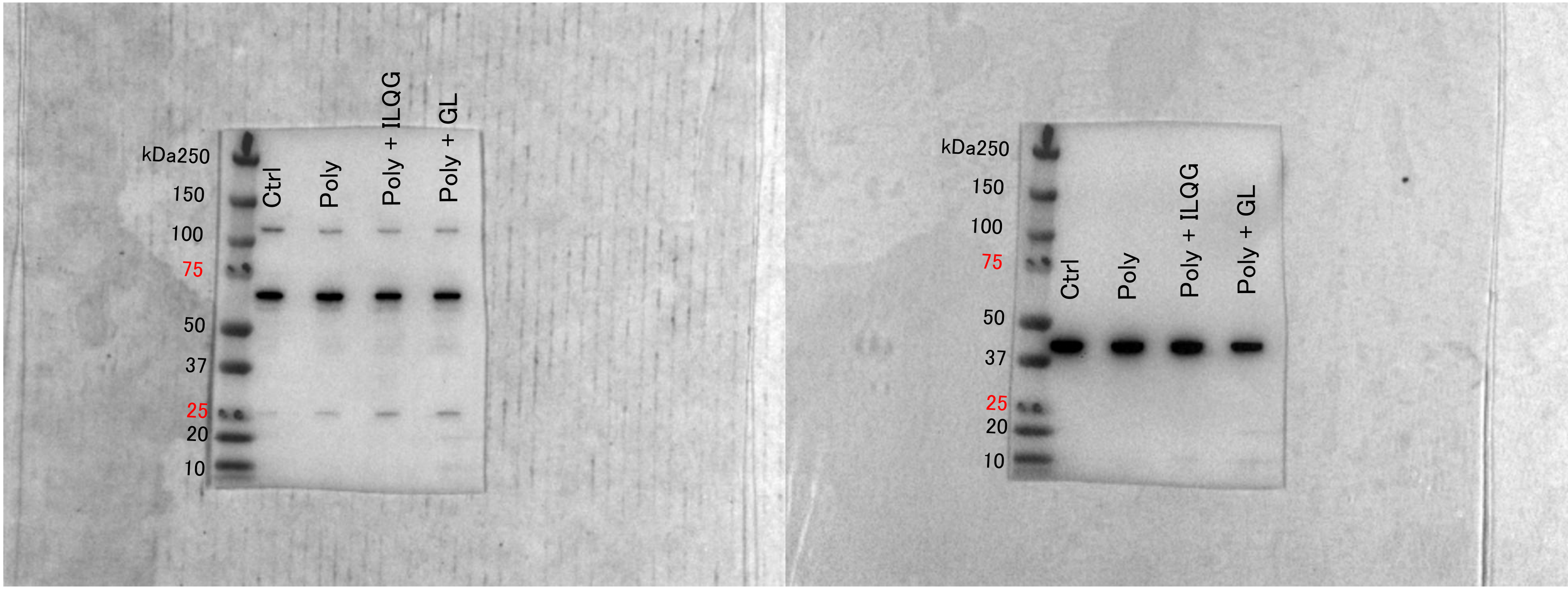

Lane assignment (from left to right):

Lane 1: Molecular weight marker
Lane 2: Control
Lane 3: Empty lane
Lane 4: Poly I:C
Lane 5: Empty lane
Lane 6: Poly I:C +ILQG
Lane 7: Empty lane
Lane 8: Poly I:C +GL

Supplementary Figure S14.
Uncropped Western blot corresponding to Figure 9 (a) (p65), Replicate 2. Target protein and β-actin were detected on the same membrane using the same protein lysates. The images show the full membranes from an independent biological replicate used for quantitative densitometric analysis presented in Figure 9 (a). All lanes are shown, including unused lanes. No brightness or contrast adjustments were applied to individual bands.

Supplementary Figure S15– p65 (Replicate 3)

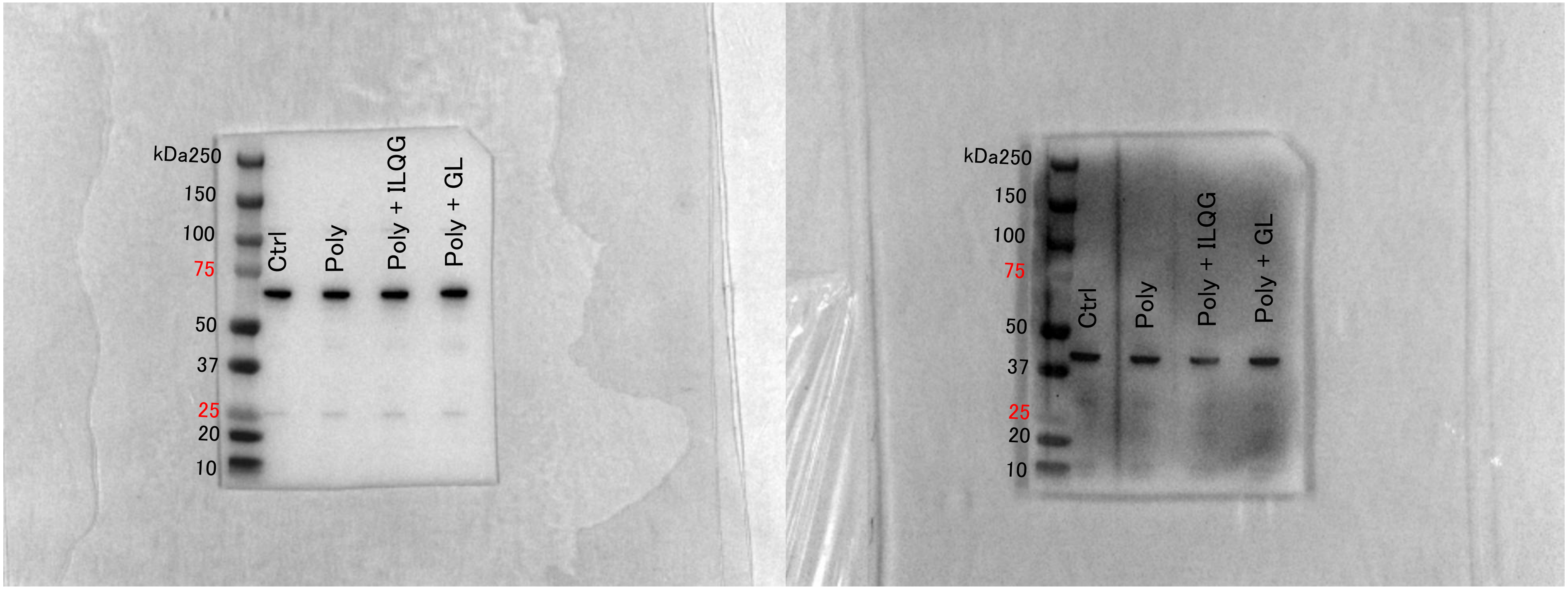

Lane assignment (from left to right):

Lane 1: Molecular weight marker
Lane 2: Control
Lane 3: Empty lane
Lane 4: Poly I:C
Lane 5: Empty lane
Lane 6: Poly I:C +ILQG
Lane 7: Empty lane
Lane 8: Poly I:C +GL

Supplementary Figure S15.
Uncropped Western blot corresponding to Figure 9 (a) (p65), Replicate 3. Target protein and β-actin were detected on the same membrane using the same protein lysates. The images show the full membranes from an independent biological replicate used for quantitative densitometric analysis presented in Figure 9 (a). All lanes are shown, including unused lanes. No brightness or contrast adjustments were applied to individual bands.

Supplementary Figure S16– P-p65 (Replicate 1)

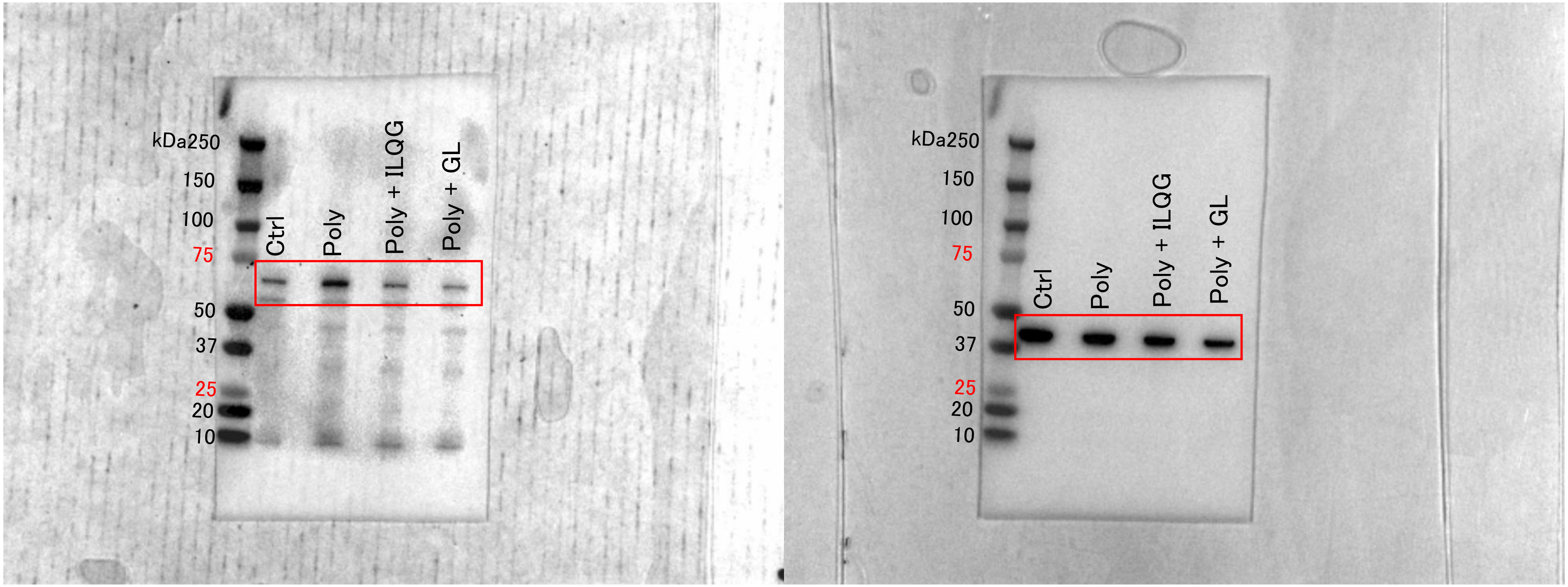

Lane assignment (from left to right):

Lane 1: Molecular weight marker
Lane 2: Control
Lane 3: Empty lane
Lane 4: Poly I:C
Lane 5: Empty lane
Lane 6: Poly I:C +ILQG
Lane 7: Empty lane
Lane 8: Poly I:C +GL

Supplementary Figure S16.
Uncropped Western blot corresponding to Figure 9 (b)(P-p65), Replicate 1.Target protein and β-actin were detected on the same membrane using the same protein lysates. The images show the full membranes used to generate the main figure. Red boxes indicate the cropped regions presented in the manuscript. All lanes are shown, including unused lanes. No brightness or contrast adjustments were applied to individual bands.

Supplementary Figure S17– P-p65 (Replicate 2)

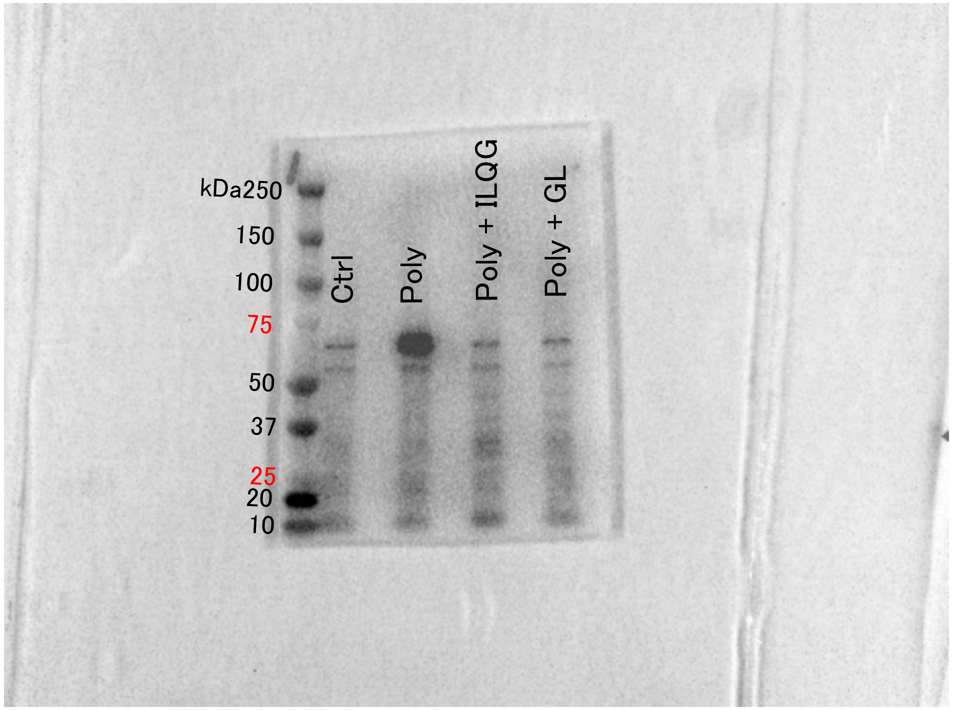

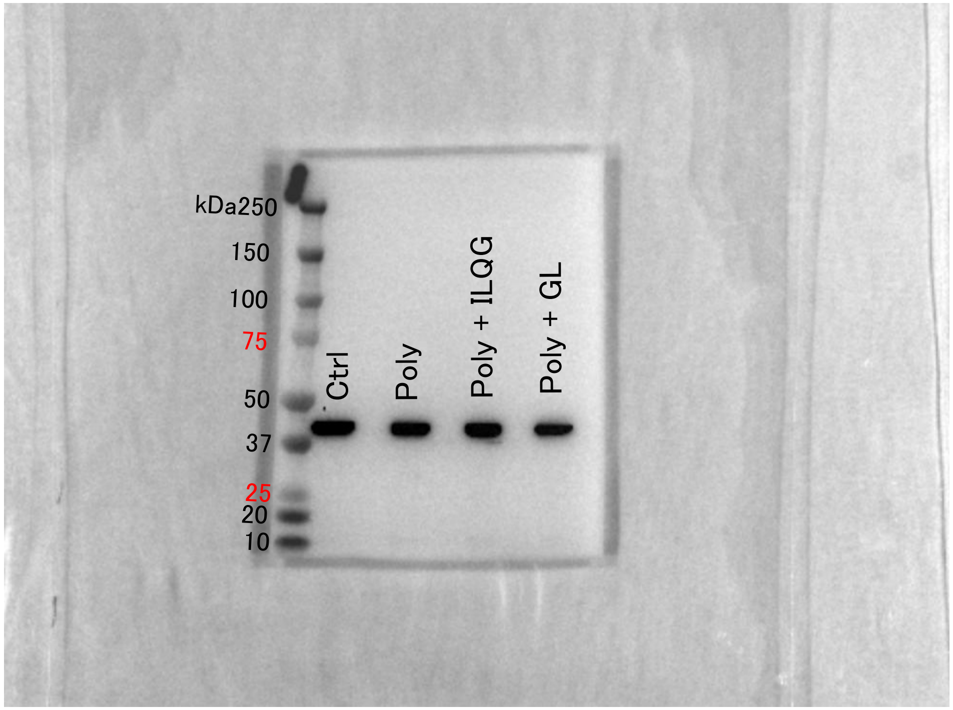

Lane assignment (from left to right):

Lane 1: Molecular weight marker
Lane 2: Control
Lane 3: Empty lane
Lane 4: Poly I:C
Lane 5: Empty lane
Lane 6: Poly I:C +ILQG
Lane 7: Empty lane
Lane 8: Poly I:C +GL

Supplementary Figure S17.
Uncropped Western blot corresponding to Figure 9 (b), (P-p65), Replicate 2. Target protein and β-actin were detected on the same membrane using the same protein lysates. The images show the full membranes from an independent biological replicate used for quantitative densitometric analysis presented in Figure 9 (b). All lanes are shown, including unused lanes. No brightness or contrast adjustments were applied to individual bands.

Supplementary Figure S18– P-p65 (Replicate 3)

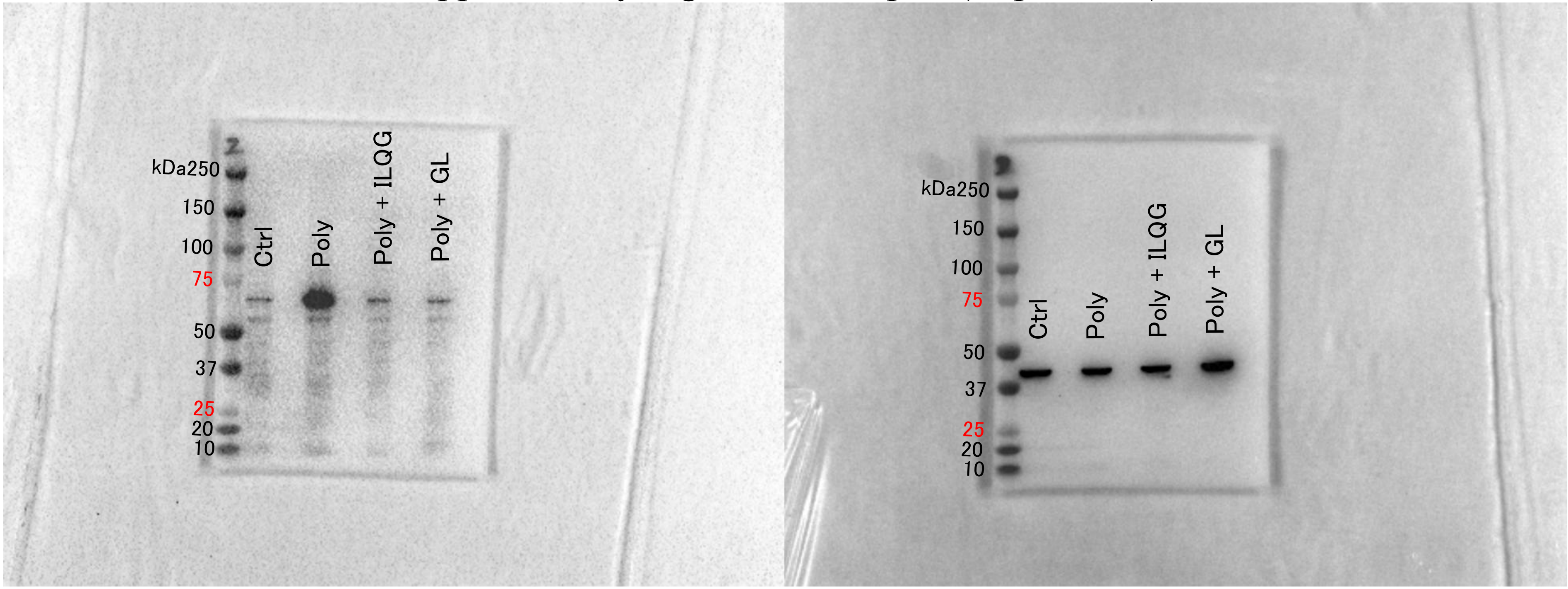

Lane assignment (from left to right):

Lane 1: Molecular weight marker
Lane 2: Control
Lane 3: Empty lane
Lane 4: Poly I:C
Lane 5: Empty lane
Lane 6: Poly I:C +ILQG
Lane 7: Empty lane
Lane 8: Poly I:C +GL

Supplementary Figure S18.
Uncropped Western blot corresponding to Figure 9 (b), (P-p65), Replicate 3. Target protein and β-actin were detected on the same membrane using the same protein lysates. The images show the full membranes from an independent biological replicate used for quantitative densitometric analysis presented in Figure 9 (b). All lanes are shown, including unused lanes. No brightness or contrast adjustments were applied to individual bands.

Supplementary Figure S19– IκBα (Replicate 1)

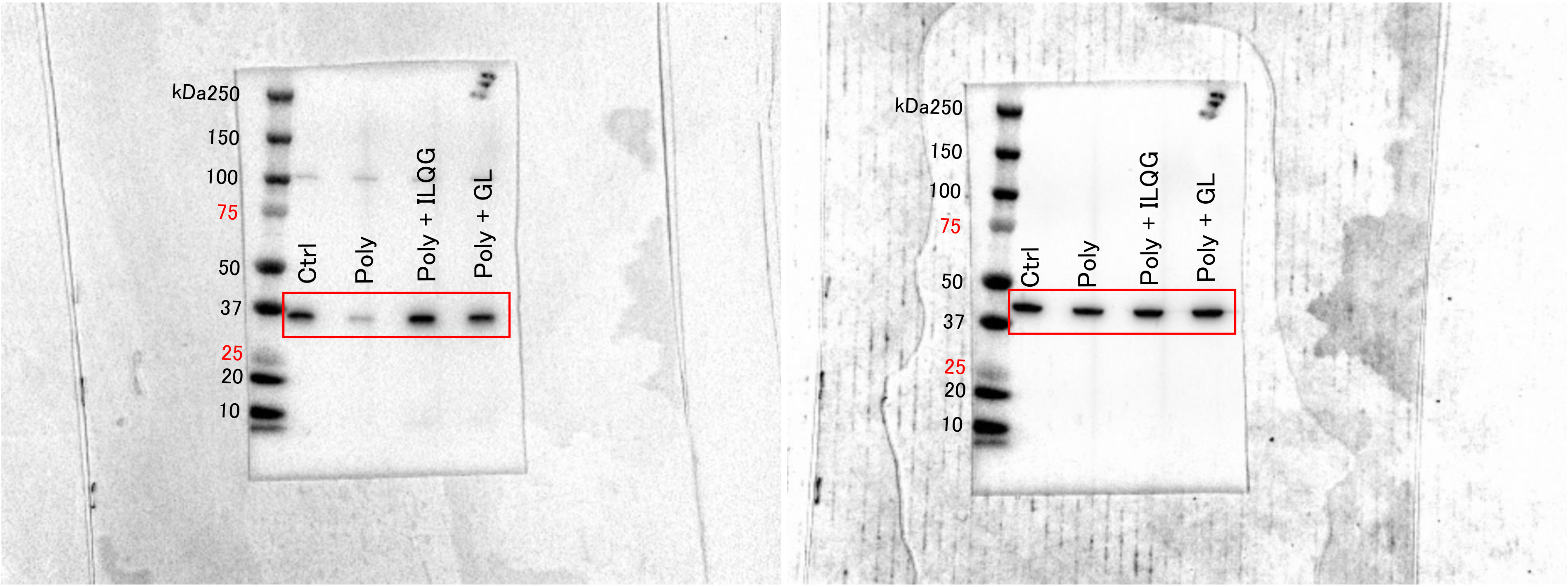

Lane assignment (from left to right):

Lane 1: Molecular weight marker
Lane 2: Control
Lane 3: Empty lane
Lane 4: Poly I:C
Lane 5: Empty lane
Lane 6: Poly I:C +ILQG
Lane 7: Empty lane
Lane 8: Poly I:C +GL

Supplementary Figure S19.
Uncropped Western blot corresponding to Figure 9 (d), (IκBα), Replicate 1.Target protein and β-actin were detected on the same membrane using the same protein lysates. The images show the full membranes used to generate the main figure. Red boxes indicate the cropped regions presented in the manuscript. All lanes are shown, including unused lanes. No brightness or contrast adjustments were applied to individual bands.

Supplementary Figure S20– IκBα (Replicate 2)

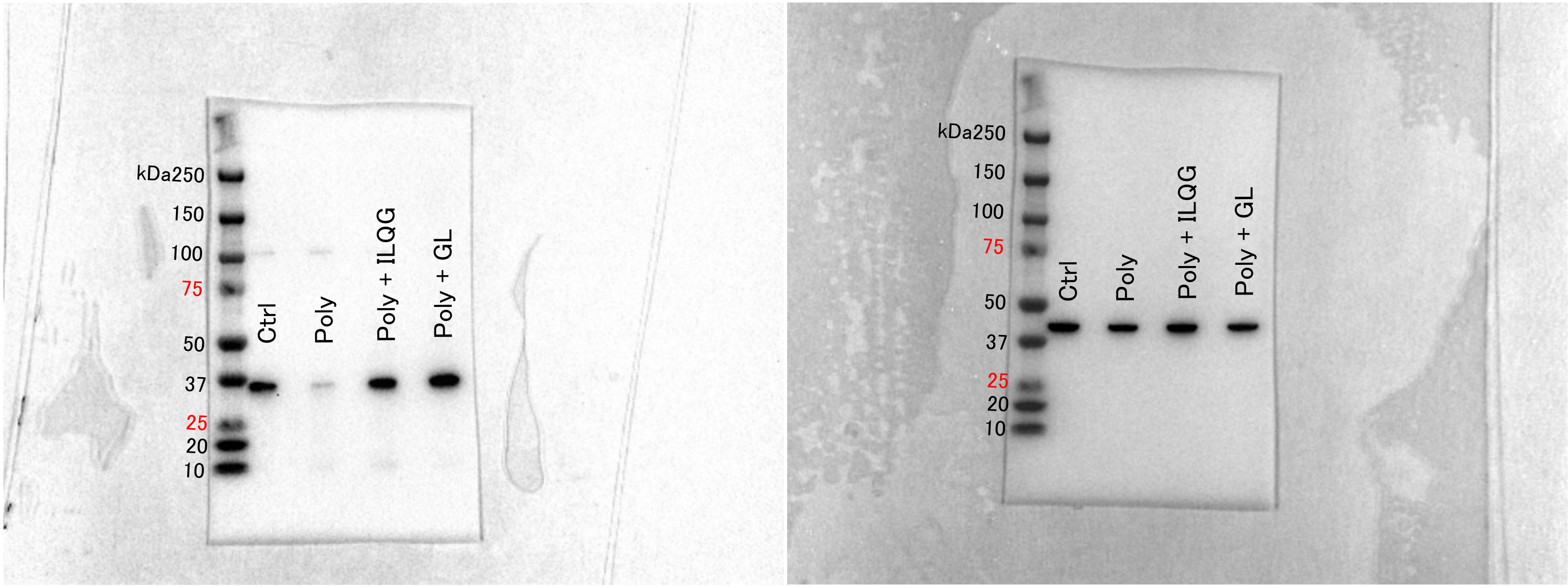

Lane assignment (from left to right):

Lane 1: Molecular weight marker
Lane 2: Control
Lane 3: Empty lane
Lane 4: Poly I:C
Lane 5: Empty lane
Lane 6: Poly I:C +ILQG
Lane 7: Empty lane
Lane 8: Poly I:C +GL

Supplementary Figure S20.
Uncropped Western blot corresponding to Figure 9 (d), (IκBα), Replicate 2. Target protein and β-actin were detected on the same membrane using the same protein lysates. The images show the full membranes from an independent biological replicate used for quantitative densitometric analysis presented in Figure 9 (d). All lanes are shown, including unused lanes. No brightness or contrast adjustments were applied to individual bands.

Supplementary Figure S21– IκBα (Replicate 3)

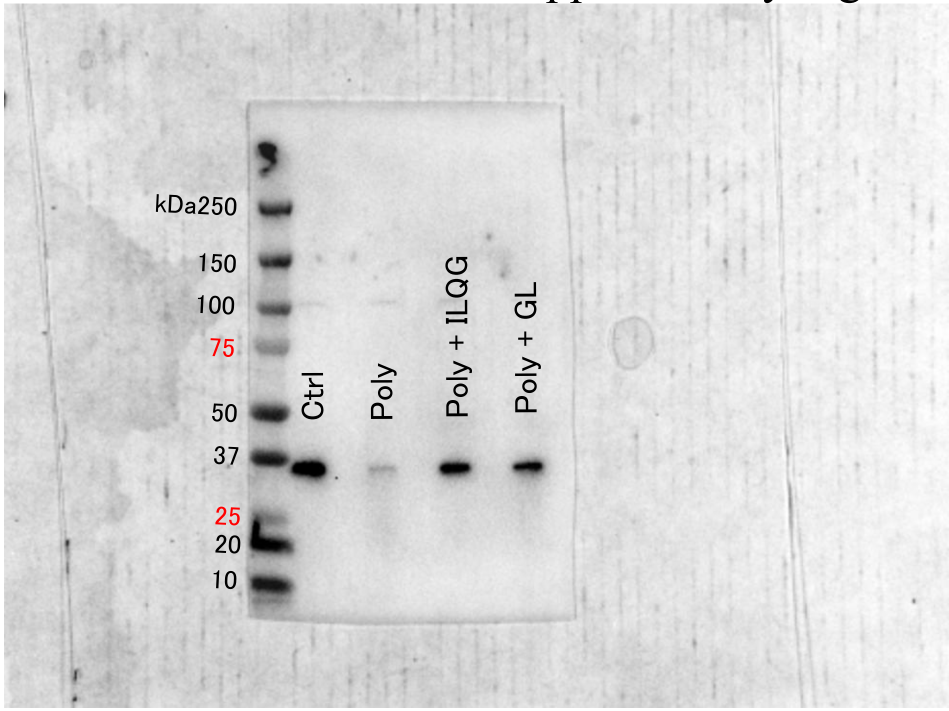

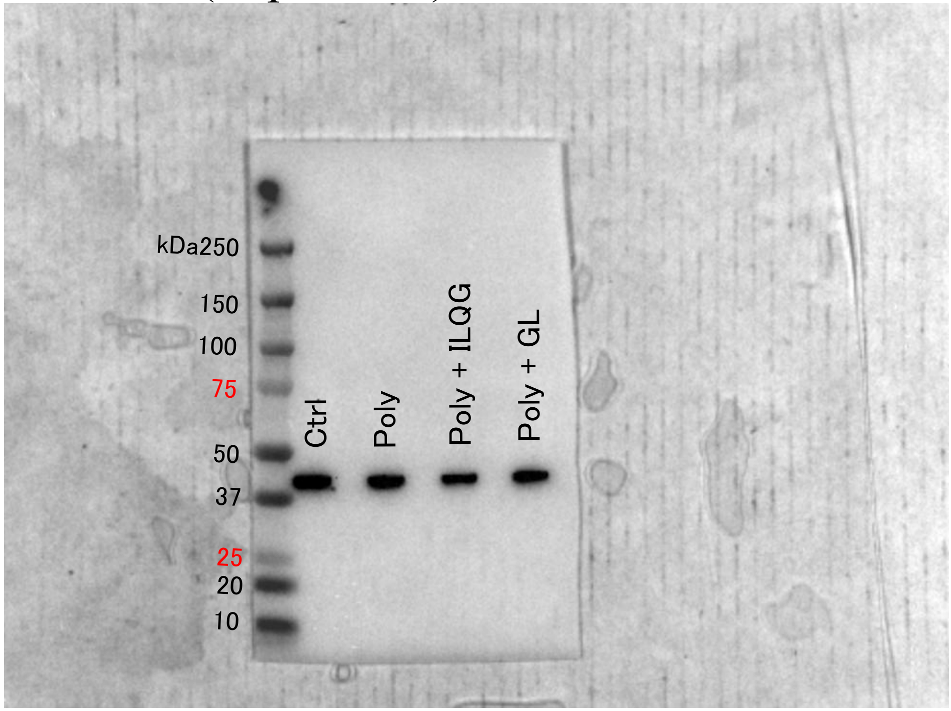

Lane assignment (from left to right):

Lane 1: Molecular weight marker
Lane 2: Control
Lane 3: Empty lane
Lane 4: Poly I:C
Lane 5: Empty lane
Lane 6: Poly I:C +ILQG
Lane 7: Empty lane
Lane 8: Poly I:C +GL

Supplementary Figure S21.
Uncropped Western blot corresponding to Figure 9 (d), (IκBα), Replicate 3. Target protein and β-actin were detected on the same membrane using the same protein lysates. The images show the full membranes from an independent biological replicate used for quantitative densitometric analysis presented in Figure 9 (d). All lanes are shown, including unused lanes. No brightness or contrast adjustments were applied to individual bands.

Supplementary Figure S22– P-IκBα (Replicate 1)

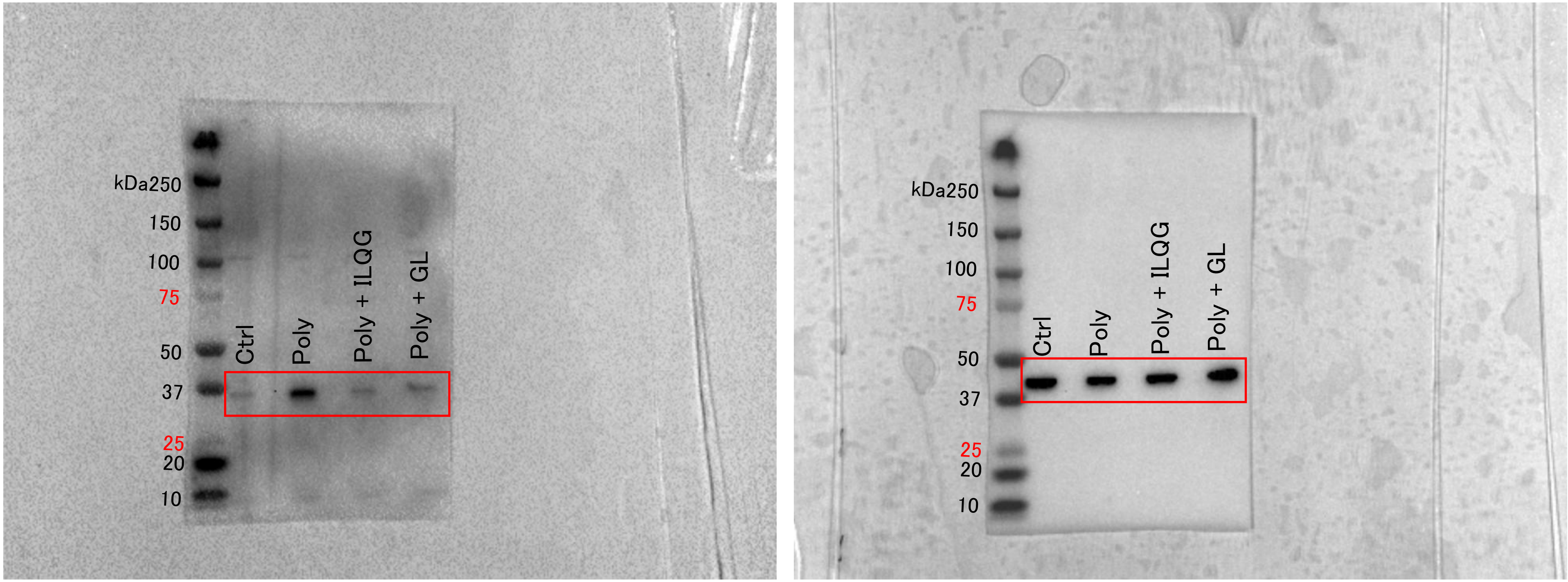

Lane assignment (from left to right):

Lane 1: Molecular weight marker
Lane 2: Control
Lane 3: Empty lane
Lane 4: Poly I:C
Lane 5: Empty lane
Lane 6: Poly I:C +ILQG
Lane 7: Empty lane
Lane 8: Poly I:C +GL

Supplementary Figure S22.
Uncropped Western blot corresponding to Figure 9 (e), (P-IκBα), Replicate 1.Target protein and β-actin were detected on the same membrane using the same protein lysates. The images show the full membranes used to generate the main figure. Red boxes indicate the cropped regions presented in the manuscript. All lanes are shown, including unused lanes. No brightness or contrast adjustments were applied to individual bands.

Supplementary Figure S23– P-IκBα (Replicate 2)

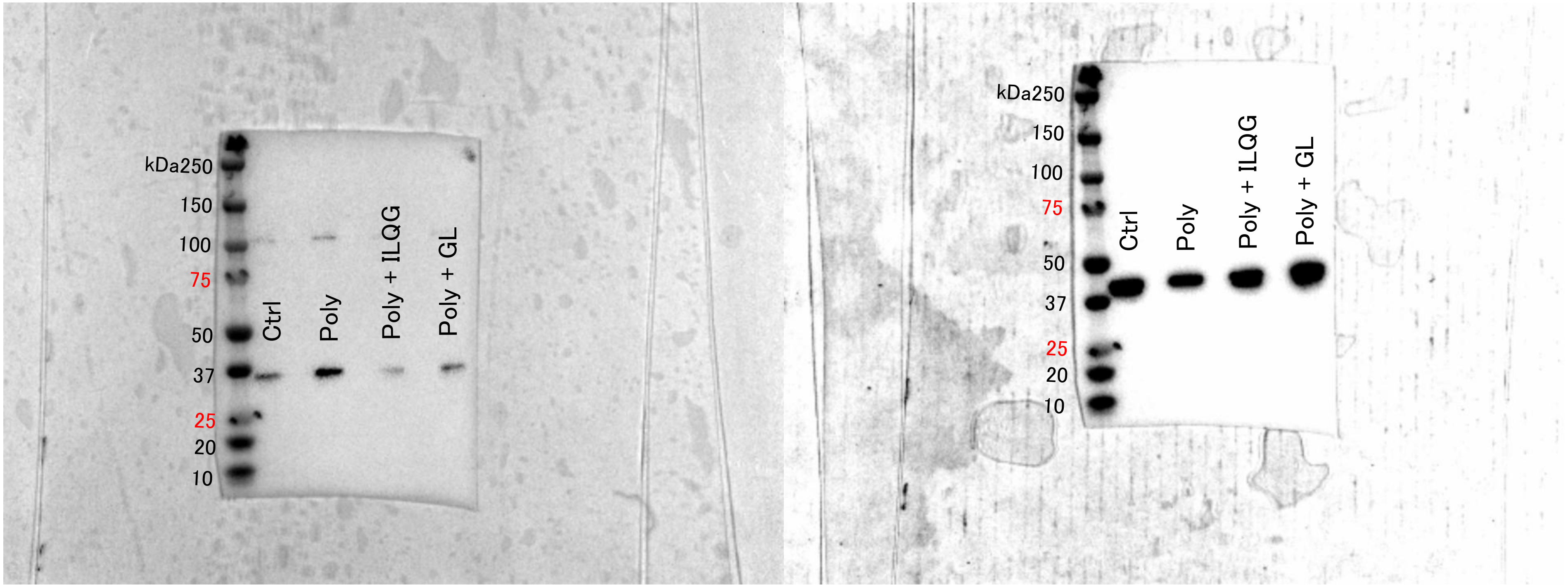

Lane assignment (from left to right):

Lane 1: Molecular weight marker
Lane 2: Control
Lane 3: Empty lane
Lane 4: Poly I:C
Lane 5: Empty lane
Lane 6: Poly I:C +ILQG
Lane 7: Empty lane
Lane 8: Poly I:C +GL

Supplementary Figure S23.
Uncropped Western blot corresponding to Figure 9 (e), (P-IκBα), Replicate 2. Target protein and β-actin were detected on the same membrane using the same protein lysates. The images show the full membranes from an independent biological replicate used for quantitative densitometric analysis presented in Figure 9 (e). All lanes are shown, including unused lanes. No brightness or contrast adjustments were applied to individual bands.

Supplementary Figure S24– P-IκBα (Replicate 3)

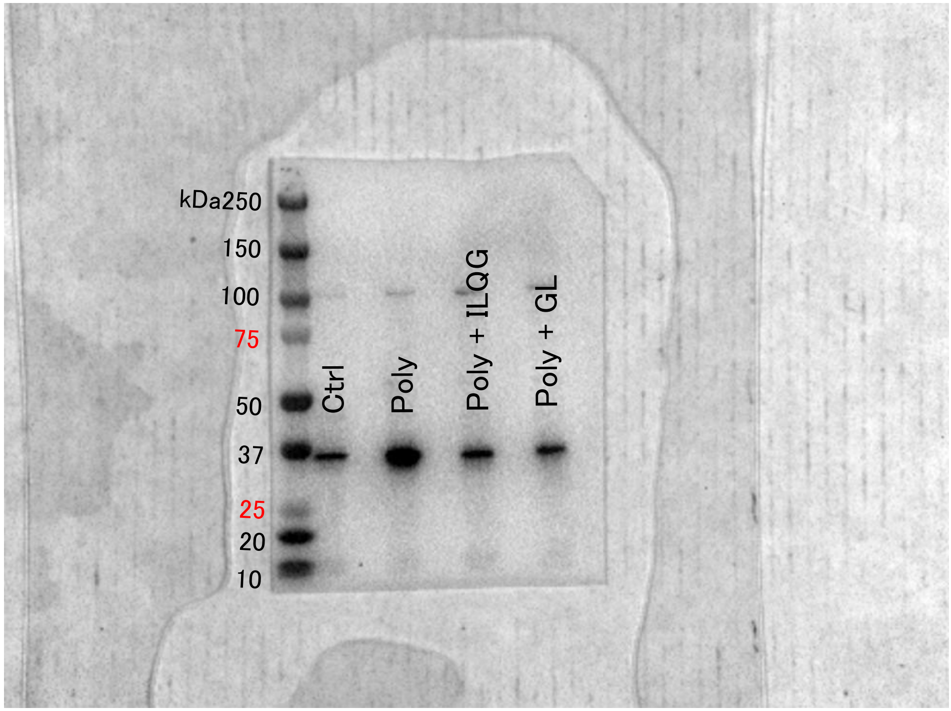

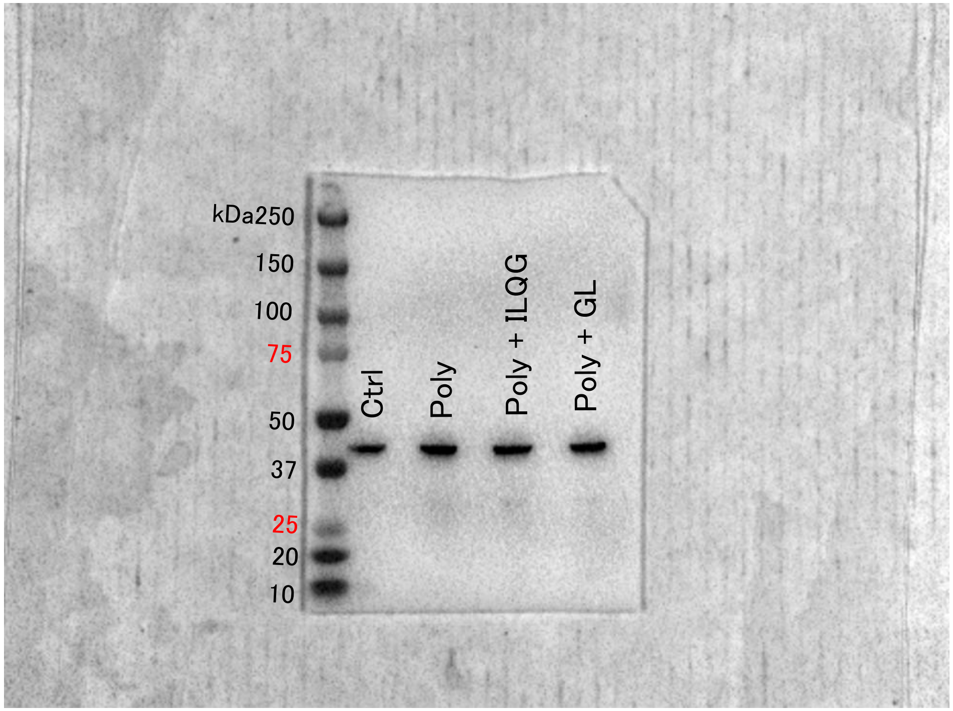

Lane assignment (from left to right):

Lane 1: Molecular weight marker
Lane 2: Control
Lane 3: Empty lane
Lane 4: Poly I:C
Lane 5: Empty lane
Lane 6: Poly I:C +ILQG
Lane 7: Empty lane
Lane 8: Poly I:C +GL

Supplementary Figure S24.
Uncropped Western blot corresponding to Figure 9 (e), (P-IκBα), Replicate 3. Target protein and β-actin were detected on the same membrane using the same protein lysates. The images show the full membranes from an independent biological replicate used for quantitative densitometric analysis presented in Figure 9 (e). All lanes are shown, including unused lanes. No brightness or contrast adjustments were applied to individual bands.
